# Gender-Specific Fine Motor Skill Learning Is Impaired by Myelin-Targeted Neurofibromatosis Type 1 Gene Mutation

**DOI:** 10.3390/cancers16030477

**Published:** 2024-01-23

**Authors:** Daniella P. Hernandez, Daniela M. Cruz, Celeste S. Martinez, Larisa M. Garcia, Ashley Figueroa, Marisol Villarreal, Liya M. Manoj, Saul Lopez, Karla D. López-Lorenzo, Alejandro López-Juárez

**Affiliations:** 1 Department of Health and Biomedical Sciences, University of Texas Rio Grande Valley, Brownsville, TX 78520, USA; 2 Department of Biology, Texas A&M University, College Station, TX 77843, USA

**Keywords:** neurofibromatosis type 1, myelin, white matter, oligodendrocytes, RASopathies

## Abstract

**Simple Summary:**

Most neurofibromatosis type 1 (NF1) patients present with neurological issues in parallel with abnormal brain white matter and myelin. Although links for NF1 neuropathophysiology and abnormal myelin were proposed long ago, no current data can openly support or refute this idea. Various studies suggest that *Nf1* mutations affect myelin biology and function, but any impact on learning/memory remains unclear. Here, we show that mice with an *Nf1* mutation induced in adult myelinating cells present learning, but not memory, issues in a voluntary fine motor skill test, the complex wheel (CW). The specific parameters shaping CW learning curves are differentially impacted in an *Nf1* mutation dose- and gender-dependent manner and are responsive to nitric oxide modulation. Fate analyses of *Nf1* mutant cells support links between defective myelin and fine motor learning issues. Our results diversify the potential therapeutic targets and windows of time for NF1 treatments that restore or improve myelin function.

**Abstract:**

Neurofibromatosis type 1 (NF1) is caused by mutations in the *NF1* gene. The clinical presentation of NF1 includes diverse neurological issues in pediatric and adult patients, ranging from learning disabilities, motor skill issues, and attention deficit disorder, to increased risk of depression and dementia. Preclinical research suggests that abnormal neuronal signaling mediates spatial learning and attention issues in NF1; however, drugs that improve phenotypes in models show inconclusive results in clinical trials, highlighting the need for a better understanding of NF1 pathophysiology and broader therapeutic options. Most NF1 patients show abnormalities in their brain white matter (WM) and myelin, and links with NF1 neuropathophysiology have been suggested; however, no current data can clearly support or refute this idea. We reported that myelin-targeted *Nf1* mutation impacts oligodendrocyte signaling, myelin ultrastructure, WM connectivity, and sensory–motor behaviors in mice; however, any impact on learning and memory remains unknown. Here, we adapted a voluntary running test—the complex wheel (CW; a wheel with unevenly spaced rungs)—to delineate fine motor skill learning curves following induction of an *Nf1* mutation in pre-existing myelinating cells (*pNf1* mice). We found that *pNf1* mutant females experience delayed or impaired learning in the CW, while proper learning in *pNf1* males is predominantly disrupted; these phenotypes add complexity to the gender-dependent learning differences in the mouse strain used. No broad differences in memory of acquired CW skills were detected in any gender, but gene-dose effects were observed at the studied time points. Finally, nitric oxide signaling regulation differentially impacted learning in wild type (WT)/*pNf1*, male/female mice. Our results provide evidence for fine motor skill learning issues upon induction of an *Nf1* mutation in mature myelinating cells. Together with previous connectivity, cellular, and molecular analyses, these results diversify the potential treatments for neurological issues in NF1.

## 1. Introduction

Neurofibromatosis type 1 (NF1) is the most common RASopathy (diseases involving mutations impacting the RAS/MAPK pathway) with prevalence of 1/2500–1/3000 [1,2] and is caused by an inherited or de novo mutation in the *NF1* gene. The protein product of *NF1*, neurofibromin, is well known for its roles in the control of cell proliferation mediated by the RAS/MAPK and linked pathways [3]. Despite its monogenic origin, the clinical presentation of NF1 is highly variable [4,5]. Most NF1 pediatric patients present with diverse, in terms of type and severity, neurological manifestations including learning disabilities, motor skill issues, and attention deficit disorder (ADD) with or without hyperactivity [6,7,8,9,10]. With age, deficits in cognitive function remain or decline [11], and new issues arise including an increased risk for depression and dementia [12,13,14,15]. The mechanisms for NF1 neurological manifestations are not clear, but significant insights have been obtained using adult mouse genetic models; spatial learning issues have been attributed to abnormal GABA signaling [16] and attention system dysfunction to striatal dopaminergic neurons [17,18]. However, drugs that improve neurological phenotypes in models have shown inconclusive results in clinical trials [19,20], which precludes final recommendations for NF1 treatment [21,22,23].

In correlation with cognitive [24], attention [25], and reaction time [26] abnormalities, NF1 pediatric and adult patients show defects in their brain’s white matter (WM) including enlarged WM tracts [27,28,29], abnormal microstructures detected by dti-MRI, and connectivity issues, putatively involving compromised myelin [30,31]. Although the involvement of defective myelin in NF1 neuropathophysiology was suggested decades ago [32], no data can openly support or refute this idea [33,34,35]. A recent surge in knowledge on myelin plasticity as a regulator of fine motor learning [36] and working memory [37] makes this idea feasible to test. As developmental and subsequent adaptive myelination improves nerve conduction speed, signal synchronization, and overall information processing in the nervous system, *Nf1* mutation-driven defects in adult oligodendrocytes (OLs, the central myelin producers) potentially impact higher-order brain functions including learning. It was suggested that *Nf1* mutations impact early myelin biology from the specification to proliferation of OL precursors, but broad myelination or myelin content show no obvious changes in NF1 patients or models (reviewed in [38]). Therefore, to unveil the effects of *Nf1* mutant myelin on learning, the scope of this study was to describe CW learning following *Nf1* mutation induction in adult myelinating cells.

In adults, neurofibromin is mostly found in cells of the nervous system [39,40,41,42,43] and an *Nf1* mutation in myelinating cells (in *PlpCre^ER^*; *Nf1flox* or *pNf1* mice) causes progressive OL myelin decompaction at ~1 month and ~6 months in homozygous and hemizygous mice, respectively [44,45]. Myelin decompaction was also found in NF1 fish [46] and pig [47] models, although to a lesser extent. Six months after adult *pNf1* mutation induction, sensory–motor issues in the pre-pulse inhibition of the startle response and rotarod tests were observed, and pharmacological inhibition of Notch or nitric oxide (NO) signaling reverted these abnormalities [44,48]. Nonetheless, the impact of the *pNf1* mutation on learning and memory has not been studied. Here, we modified a myelination-regulated fine motor skill learning test, the complex wheel (CW; voluntary running on a wheel with unevenly spaced rungs), to study the impact of *Nf1* mutations in pre-existing myelinating cells on learning and memory. First, we identified previously unrecognized gender-related differences in CW skill learning in WT mice. Second, learning curves for most CW parameters showed delays in homozygous and disruption in hemizygous *pNf1* female mutants. Third, CW skill learning was consistently subnormal in both homozygous and hemizygous *pNf1* male mutants. Lastly, nitric oxide signaling differentially modulated learning in WT and in *pNf1* males/females. Our study provides evidence of the impact of *Nf1* mutations in mature myelinating cells on the control of fine motor skill learning. The CW test applied to NF1 models will expedite mechanistic studies, as well as the screening of treatments aimed at improving myelin function and motor learning in NF1.

## 2. Materials and Methods

### 2.1. Animals

All mouse studies were approved by the University of Texas Rio Grande Valley Institutional Animal Care and Use Review Committee (IACUC). Validation of mouse strains and selection of alleles of interest were performed by genotyping, as previously described for *Nf1^FLOX^* [49], *PlpCre^ERT2^* [50], and *cag-catEGFP* [51] alleles. The mouse genotypes used were *PlpCre^ERT2^*; *Nf1^FLOX/+^* (henceforth ***pNf1 f/+***), *PlpCre^ERT2^*; *Nf1^FLOX/FLOX^* (henceforth ***pNf1f/f***), and *PlpCre^ERT2^*; *Nf1^+/+^*, *PlpCre^ERT2^,* or *Nf1^FLOX/+^* (phenotypically **WT** and indistinguishable from each other). The Cre^ERT2^-inducible EGFP reporter gene *cag-catEGFP* was utilized in combination with *pNf1 f/+*, *pNf1 f/f*, and WT alleles to study the dynamics of recombinant cells. All alleles were back crossed for >10 generations with the *C57BL/6J* strain background. Studies in NF1 patients and models show gender-dependent neurological issues [52,53,54]; hence, female and male mice were independently assessed and analyzed. Mice were housed in a temperature- and humidity-controlled vivarium on a 12 h light–dark cycle with free access to food and water. Body weight was measured in all individuals before tamoxifen treatment, at day 1 running in CWs, day 36 (reintroduction to CWs), and at the end of CW test (day 42). *pNf1 f/f* mice (but not *pNf1 f/+*) show phenotypes at 1-month post-mutation induction (p-m), both *pNf1 f/+* and *pNf1 f/f* mice show phenotypes at 6 months p-m [42,46], but *pNf1 f/f* mice develop nerve tumors at 5 months p-m [55]; hence, we analyzed *pNf1 f/f* mice 2–4 months and *pNf1 f+* mice 2–6 months p-m.

### 2.2. Tamoxifen and L-NAME Treatment

To induce recombination/mutation of *Nf1* in myelinating cells and expression of the reporter gene EGFP, adult mice (2 months old; 2 MO) were injected intraperitoneally with tamoxifen (75 mg/kg of body weight, in sunflower seed oil; Sigma-Aldrich, St. Louis, MI, USA) twice daily for 3 consecutive days. The nitric oxide synthase 1–3 inhibitor N-nitroarginine methyl ester (L-NAME, Millipore Sigma; Burlington, MA, USA) was dissolved in drinking water at 0.3 g/L and stored at 4 °C protected from light. Mice were subjected to oral L-NAME treatment (fed ad libitum) for 4 days before and during the CW test. The L-NAME water was replaced every 48 h.

### 2.3. Complex Wheel (CW) Learning/Memory Test

In short, mice were introduced to the CWs for 2 weeks, housed without the wheel for 3 weeks, and reintroduced to the CWs for 1 week. Specifically, at 2–6 months p-m, mice were introduced to wheels with 22 unevenly spaced rungs at the beginning of the 12 h dark cycle, and running activity was continuously recorded by an infrared system coupled to dedicated software (SAS, Lafayette Instruments, Lafayette, IN, USA). Running time bin size was set to meters per minute and analyses of parameters (see statistics) were performed for each 12 h dark period (nights), as well as for each 15 min interval in single nights (48 intervals/night). Light intensity was homogeneous among cages and cage location was randomized to avoid effects of cage position. The mice’s whiskers were trimmed on day 1 of the CW test to avoid detection of missing rungs with whiskers and to avoid the influence of potential defects in whisker sensitivity; abnormal barrel formation in the somatosensory cortex was reported in NF1 mouse models [56].

### 2.4. Brain Dissection, Immunostaining, and Imaging

Mice were overdosed with anesthesia and perfused with 1X PBS followed by ice cold 4% paraformaldehyde. Brains were dissected, fixed in 4% paraformaldehyde overnight, and sectioned using a vibratome (Leica, Wetzlar, Germany). Floating sections were processed for immunodetection of cell markers using specific antibodies for GFP (Nacalai Tesque, Kyoto, Japan), CC1 (Calbiochem, San Diego, CA, USA), NG2 (Millipore, Burlington, MA, USA), Olig2 (Millipore), and NeuN (Millipore). Fluorophore-conjugated (Cy2, Cy3, and Cy5; Jackson ImmunoResearch, West Grove, PA, USA) secondary antibodies were used to detect the antigen–antibody complexes. Using the appropriate laser excitation wavelengths for the secondary antibodies, images were collected using a Leica 2500SPE confocal microscope. Automated and manual (for NG2 staining) cell counting was performed using ImageJ and the ITCN plugin. Specifically, an area/region of interest of 1 mm^2^ (Arbitrary Units; AU) was established for all confocal images and the total number of marker+ cells was automatically calculated using the same ImageJ/ITCN (NIH, MD) parameters for size, distance between cells, and signal threshold per marker in all images.

### 2.5. Data Analyses and Statistics

CW raw data obtained with SAS software Version 23.05 (Lafayette Instruments, IN) for distance run/minute (time bin size) during the entire test (2 weeks + 1 week after a break period) were processed by investigators blinded to the genotypes. Five parameters were obtained from each 12 h dark period or from the 15 min intervals from specific single nights. The total distance (meters) per period and Total Average Speed (TAS; average meters/min, considering all minutes/period) are broad measurements of CW performance; the maximum speed corresponds to the highest speed achieved per period (m/min); the Activity Average Speed (AAS) is the average speed of all minutes running >1 m (0 values excluded); and the activity corresponds to the number of minutes running >1 m per period/interval. The CW average speed of phenotypically WT mice has an approximately normal distribution (*p* = 0.2, K-S test) that, together with the low data variability, evidence significant differences using a low number of mice [36,57]. To account for the potential impact of phenotype variability caused by the *Nf1* mutation, we used a minimum “n” of 5 mice/gender/genotype. All quantifications are presented as mean +/− standard error of the mean. Data were analyzed by two-way ANOVA with gender, genotype, or time as sources of variation, and single-night comparisons between WTs vs. mutants were performed using Bonferroni’s post hoc tests (*p* < 0.05). Slope comparisons and intercept/elevation (if slopes were not significantly different) were performed using two-tailed simple linear regression (*p* < 0.05). Unpaired Student’s *t*-tests were used for specific two-group comparisons of immunostainings (* *p* < 0.05). All statistical analyses were performed using Prism 9 software (GraphPad, Boston, MA).

## 3. Results

### 3.1. Gender-Specific Complex Wheel Performance in WT Mice

**Figure 1:**
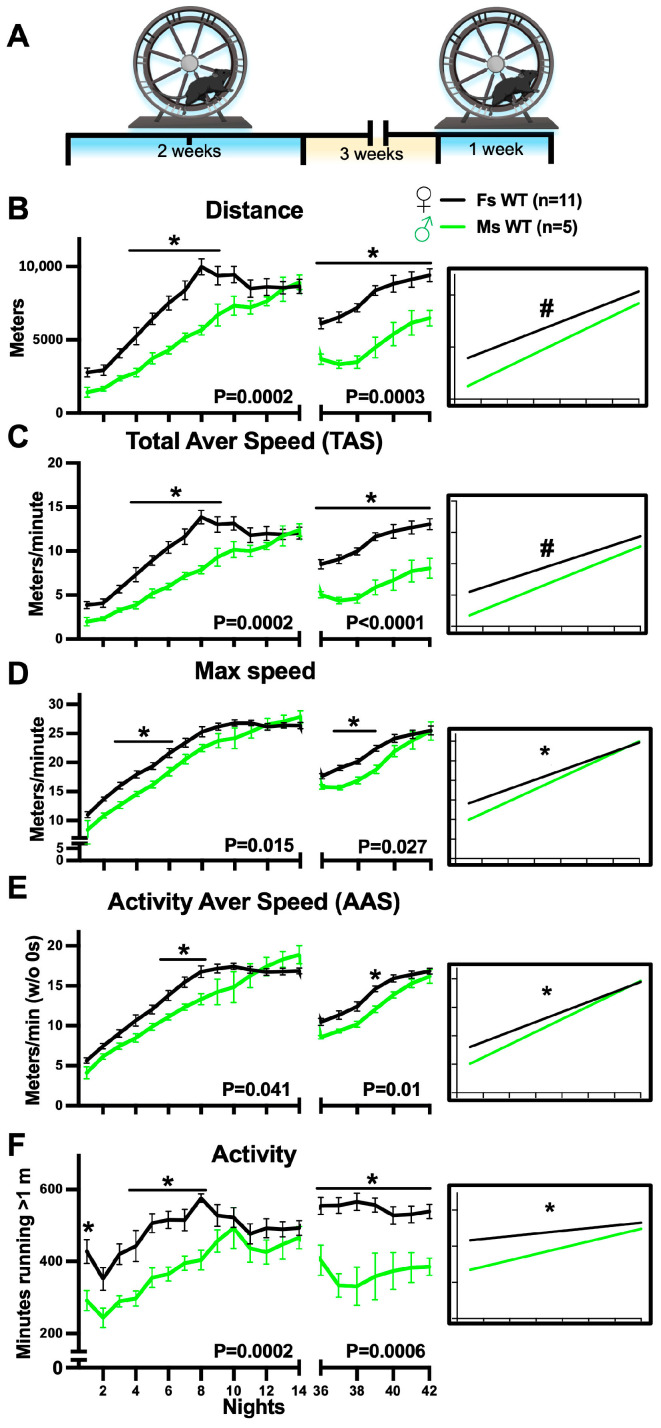
Gender-dependent differences in CW performance in WT mice. X axis labels are shared in (**B**–**F**). (**A**) CW learning/memory test: individual mice were introduced to cages with CWs for 2 weeks (blue, left), housed without wheels for 3 weeks (gap), and re-introduced to CWs for 1 additional week (blue, right). (**B**–**F**) Plots for nightly (12 h dark period) values of CW parameters for wild-type (WT) female (black) and male (green) mice: (**B**) total distance run (meters), (**C**) Total Average Speed (TAS; average speed of all minutes per night), (**D**) max speed (maximum speed in meters/minute achieved per night), (**E**) Activity Average Speed (AAS, average speed of minutes running >1 m), and (**F**) activity (number of minutes running >1 m). Only statistically significant *p* values (two-way ANOVA test, gender as source of variation, females n = 11 and males n = 5) are shown under the plots for the first (nights 1–14, left) or second (nights 36–42, right) introduction to CWs. Comparison of individual nights in female vs. male are also shown (Bonferroni’s post hoc tests, * *p* < 0.05). Insets: statistical comparison of slopes and intercept/elevation of slopes (* *p* < 0.05 and # *p* < 0.05, respectively, two-tailed linear regression) between female and male mice for the first introduction to CWs.

No gender-related differences were previously detected in mice subjected to the myelination-regulated learning test CW [36,57]; however, gender-driven differences have been reported for various strains running in regular wheels (RW) [58,59]. Therefore, we first assessed the CW performance of phenotypically WT (*PlpCre^ERT2^*; *Nf1^+/+^*, *PlpCre^ERT2^*, or *Nf1^f/+^*) [44,45,55] congenic female and male mice. To function as controls for the CreER-mediated mutation, mice were treated with tamoxifen at 2 months old (2 MO) and introduced to CWs 2–6 months later, concurring with the period when *pNf1* mutant mice develop structural and functional myelin phenotypes [44,48]. The CW test protocol consisted of a learning period in which individual mice were introduced to cages with CWs for 2 weeks, then the mice were housed without wheels for 3 weeks, and the memory of acquired skills was assessed by re-introducing the mice to the CWs for an additional week (Figure 1A). The data from nights 1–14 (N1–14) and N36–42 (first and second introduction, respectively) were then analyzed. As shown in Figure 1B, the average distance run by females per night progressively increased and reached its peak on N8 (9965.5 +/− 1654.6 m) with no significant changes up to N14 (end of the first CW introduction). In males, the average distance run per night progressively increased to reach its peak on N14 (8924.5 +/− 1016.1 m). As the peak distance achieved by males and females in two weeks was not significantly different (N8 in females vs. N14 in males, unpaired Student’s *t*-test, *p* = 0.27), it is suggested that WT male and female mice develop running skills to achieve similar distances in the CW but with different learning curves. In support of this idea, a statistical comparison of all the nights of the first CW introduction indicated significant differences in distance achieved by males compared with females (two-way ANOVA; *p* = 0.0002; gender as source of variation), and single-night comparisons indicated differences for N4–9 (Figure 1B, multiple comparisons, Bonferroni’s post hoc tests, *p* < 0.05). Furthermore, a linear regression analysis of N1–14 (Figure 1B, inset) indicated that, while the slopes in both genders were not significantly different (two-tailed simple linear regression *p* = 0.0608), the slope elevation was higher in females (*p* < 0.0001), i.e., there were no apparent gender-driven differences in the rate for increasing distance, but males consistently achieved shorter distances during the first 2 weeks. Of note, the best fit curve for the different CW parameters may differ in females and males, and therefore, direct comparisons can be not fully descriptive of the phenotype. To investigate the permanence of acquired skills since the first CW introduction, we examined whether the distance achieved on N14 (highest values in both genders) was different than that achieved after a 3-week break without CWs (N36). Both female and male WT mice showed significantly decreased distance on N36 (reintroduction to CWs) compared with their respective N14 distances; nonetheless, these values were significantly higher than their corresponding N1 values (Supplementary Figure S1B,C; N1 vs. 36 and N14 vs. N36; Bonferroni’s post hoc tests, *p* < 0.05). Therefore, while both male and female WT mice were not able to achieve their peak distance right after a 3-week break, this period did not deplete the skills acquired during the first CW introduction. Finally, the overall distance run by males during the reintroduction to the CWs (N36–42) was remarkably lower compared with that of females (Figure 1B right, two-way ANOVA, *p* = 0.0003; gender as source of variation); comparisons of individual nights showed lower values for all nights (two-way ANOVA, multiple comparison Bonferroni’s post hoc tests). Taken together, these results indicate robust gender-related differences in learning curves for CW distance in WT mice.

The distance run in CWs is influenced by parameters including the Total Average Speed (TAS; average speed considering all minutes per night), the maximum speed achieved every night (max speed/night), the average speed of periods with running per night or Activity Average Speed (AAS; excludes minutes with no activity), and the level of activity (number of minutes spent running per night). To better understand the factors influencing the learning curves for the distance achieved, these parameters were individually analyzed. As shown in Figure 1C, the plot for TAS and its statistical analyses resemble those for distance, yielding essentially the same information on gender-related differences. In contrast, the data for max speed, AAS, and activity per night yielded distinctive information. The max speed achieved per night (Figure 1D) showed remarkably similar increase rates in both genders; however, a modest but statistically significant higher speed was observed in females for the first and second introduction to the CWs (two-way ANOVA, *p* = 0.015 and *p* = 0.027, respectively); particular differences were detected for N3–6 and N37–39 (two-way ANOVA, multiple comparison Bonferroni’s post hoc tests). A slope analysis of N1–14 showed significant differences in female vs. male curves (two-tailed simple linear regression, *p* < 0.05). Similar to the distance analysis, the max speed achieved by females and males on N36 (reintroduction to CWs) was significantly lower than their corresponding N14 values but higher than their N1 values (Supplementary Figure S1D,E; Bonferroni’s post hoc tests, *p* < 0.05), supporting the idea of a partial preservation of acquired skills in WT mice. Next, analyses of the AAS showed modest changes in females vs. males (two-way ANOVA, *p* = 0.041) with significantly lower values only for N6–8 and N39 (multiple comparisons; Bonferroni’s post hoc tests) as well as significantly different slopes (Figure 1E, inset). A partial preservation of the AAS was also observed in both genders (Supplementary Figure S1F,G). Overall, gender differences in max speed and AAS curves are modest with fewer nights showing differences compared with the distance and TAS results; therefore, the max speed and AAS are modest modifiers of the marked gender-related differences in WT mice.

Finally, we assessed the level of activity per night in WT mice. The activity values showed the most variability (see error bars) among all parameters analyzed; a robust gender influence was still detected for both the first and second introduction to the CWs (Figure 1F, two-way ANOVA; *p* = 0.0002 and *p* = 0.0006, respectively; gender as source of variation) and the slope comparison also showed significant differences (Figure 1F inset). We observed that males were significantly less active during N1, N4–8, and N36–42 compared with females (multiple comparisons; Bonferroni’s post hoc tests). The comparison of activity levels for N14 vs. N36 did not show significant differences in males or females (Supplementary Figure S1H,I), suggesting that both genders have the same drive/capacity to run when re-introduced to CWs. However, females (but not males) showed higher activity levels on N36 vs. N1 (two-way ANOVA, multiple comparison Bonferroni’s post hoc tests, *p* = 0.014), suggesting an influence of previous experience on the drive/capacity to run on CWs. Overall, WT female mice show significantly higher activity than males for most nights of the CW experiment; therefore, the activity level is a main contributor to gender-related differences in CW learning curves.

### 3.2. Performance of pNf1 Mice in the CW Test Is Not Impacted by Tumor Formation or Abnormal Body Weight and Nocturnal Behavior

Oligodendrocyte signaling, myelin ultrastructure, and brain connectivity are defective in homozygous (*PlpCre^ER^*; *Nf1flox/flox* or ***pNf1f/f***) and hemizygous (*PlpCre^ER^*; *Nf1flox/+ or **pNf1f/+***) *Nf1* mutant mice, 1 and 6 months post-mutation induction, respectively [44,45,48]. Of note, *pNf1f/f* mice develop neurofibromas and lethality 5 to 13 months post-mutation induction [55]. Hence, to study the impact of the *pNf1* mutation on the acquisition of fine motor skills while limiting the influence of tumor development, we introduced *pNf1f/f* and *pNf1f/+* mice to CWs 2–4 and 2–6 months after mutation induction, respectively. Mouse health was monitored during and after (post-mortem) the CW tests; one *pNf1f/f* mutant female developed a peripheral nerve tumor and was excluded from the CW analyses. As abnormal body weight (BW) could relate to disease and its variation could impact CW performance, the BW was recorded before mutation induction at 2 MO, at the first and second introduction to the CWs, and at the end of the CW tests. As shown in Supplementary Figure S2A, *pNf1* female mutants did not show significant differences in BW at any time point compared with WT mice (two-way ANOVA, post hoc Bonferroni’s multiple comparison test, adjusted *p* > 0.05). Hemizygous *pNf1* male mutants showed a modest but significant increase in BW at the time of the first introduction to CWs (two-way ANOVA, post hoc Bonferroni’s multiple comparison test, adjusted *p* = 0.047) and homozygous males did not show any significant differences. It was reported that NF1 patients can present with sleep disorders [54,60,61] which, if present in *pNf1* mice, could impact the nocturnal running behavior in the CW. As shown in Supplementary Figure S3, most CW running activity in both WT and *pNf1* mice was performed during the 12 h dark cycles, suggesting that the *Nf1* mutation in myelinating cells does not affect nocturnal running activity. Overall, it is less likely that in our CW experimental conditions, tumor formation, considerable changes in BW, or abnormal diurnal activity impacts the skill learning curves in *pNf1* mice.

### 3.3. Delayed or Moderately Decreased Fine Motor Skill Acquisition following Myelin Nf1 Mutation in Female Mice

**Figure 2:**
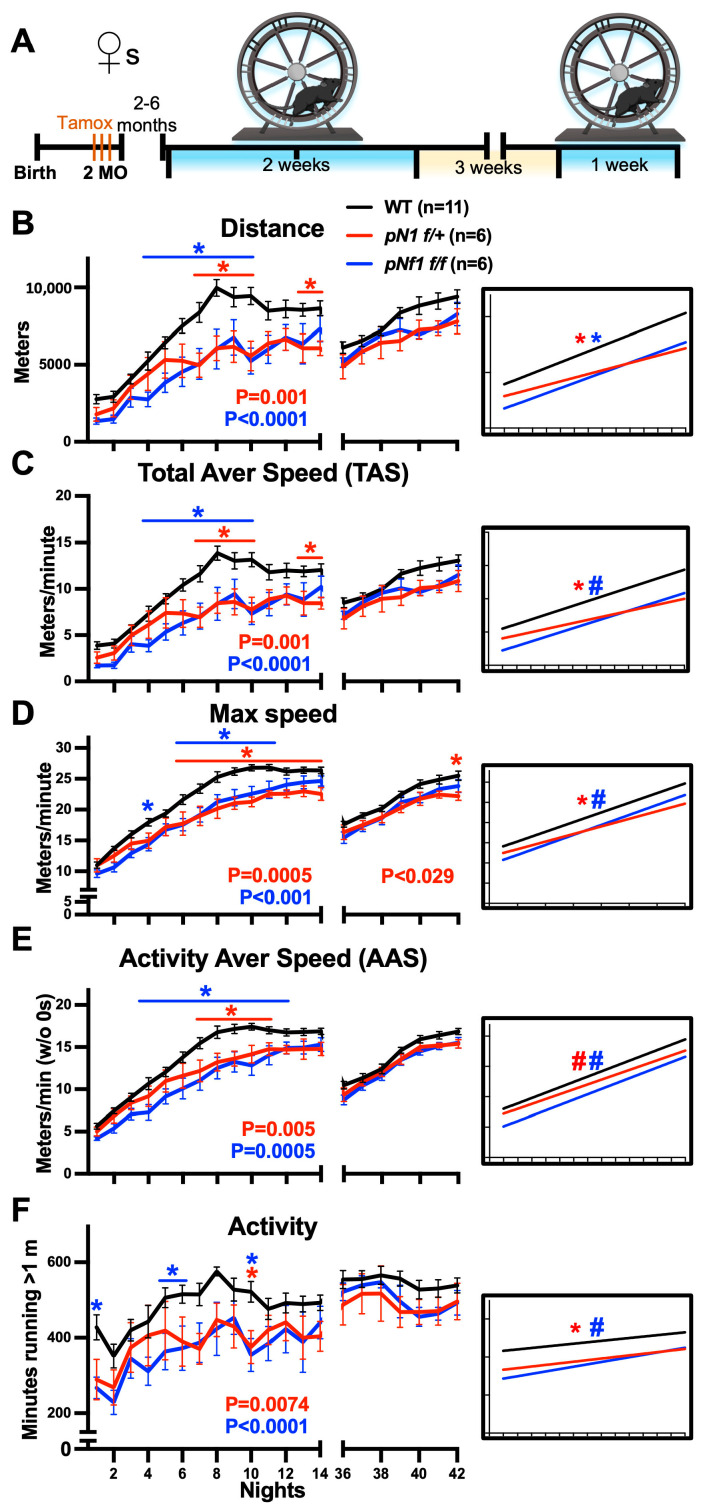
*pNf1* mutation in females delays fine motor skill acquisition. X axis labels are shared in (**B**–**F**). (**A**) Experimental protocol: 2-month-old (2 MO) WT, *pNf1f/+*, and *pNf1f/f* mice were treated with tamoxifen and 2–6 months later were introduced to CWs for 2 weeks (blue, left), housed without CW for 3 weeks (gap), and re-introduced to CWs for 1 week (blue, right). (**B**–**F**) Nightly values for CW parameters for wild-type (black), *pNf1f/+* (red), and *pNf1f/f* (blue) mice are plotted: (**B**) total distance run, (**C**) Total Average Speed (TAS), (**D**) max speed, (**E**) Activity Average Speed (AAS), and (**F**) activity. Significant *p* values from two-way ANOVA tests (genotype as source of variation; WT n = 11; *pNf1f/+* n = 6; *pNf1f/f* n = 6) are shown under plots for the first (nights 1–14, left) or second (nights 36–42, right) introduction to CWs. Individual night comparisons for WT vs. *pNf1f/+* and vs. *pNf1f/f* (Bonferroni’s post hoc tests, * *p* < 0.05) are shown. Insets: comparison of slopes and intercept/elevation of slopes for WT vs. *pNf1f/+* and vs. *pNf1f/f* are shown (two-tailed linear regression, * *p* < 0.05, and # *p* < 0.05, respectively).

Learning of fine motor skills in the CW is regulated by myelination [36,57], but any role of mature/pre-existing myelin in this process is unknown. As the central myelin structure is disrupted in *pNf1* mice [44] ~1 and ~6 months post-mutation induction in homozygotes and hemizygotes, respectively, we treated 2 MO male and female *pNf1f/+* and *pNf1f/f* mice with tamoxifen and subjected them to the CW test 2–4 and 2–6 months later, respectively (Figure 2A). Both *pNf1* hemizygous and homozygous female mutants showed significant differences in learning curves for the distance reached per night in the first introduction to the CWs compared with WTs (Figure 2B, two-way ANOVA; *p* < 0.001 for *pNf1f/+* and *p* < 0.0001 for *pNf1f/f*, genotype as source of variability). The distance run was significantly lower on N7–10 and 13–14 in *pNf1f/+*, and on N4–10 in *pNf1f/f* female mutants (multiple comparison Bonferroni’s post hoc tests). This suggests that *pNf1* females have issues developing skills to run greater distances; *pNf1f/+* mice do not achieve peak distances in two weeks as the WTs did, while *pNf1f/f* mice eventually reach peak distances on N11–14. In support of these observations, linear regression analyses indicated that the slopes for distance were significantly different in *pNf1f/+* and *pNf1f/f* mice compared with WTs (Figure 2B, inset * *p* = 0.023 and 0.025, respectively). Interestingly, during the reintroduction to the CWs (N36–42), the nightly distance reached by both *pNf1* mutants was not different than that of WTs, suggesting that the memory of the acquired skills is not affected by the *pNf1* mutation. Indeed, the distance run by *pNf1f/f* females on N36 (reintroduction to CWs) was higher than that on N1 and not different from that on N14 (Supplementary Figure S1B, two-way ANOVA, Bonferroni’s post hoc tests). No differences between N36 vs. N1 or N14 were detected in *pNf1f/+*, likely due to issues with learning the skills and the high data variability. The results for TAS were similar to those for distance: *pNf1f/+* and *pNf1f/f* females showed significant differences for the first (two-way ANOVA; *p* < 0.001 and *p* < 0.0001, respectively) but not second introduction to the CWs compared to WTs (Figure 2C). Overall, these data suggest a delayed (*pNf1f/f*) or impaired (*pNf1f/+*) skill acquisition, impacting learning the curves for CW distance and TAS in mutant female mice.

The curve for max speed/per night achieved during the first CW introduction was significantly lower in both *pNf1* mutant females (two-way ANOVA; *p* = 0.0005 in *pNf1f/+* and *p* < 0.001 in *pNf1f/f*, genotype as source of variability) and the slope and elevation of the slope were different for *pNf1f+* and *pNf1f/f*, respectively, compared to the WTs (Figure 2D, inset, two-tailed simple linear regression). Modest but significantly decreased max speeds were found on N6–14 and N4, 6–11 in *pNf1f/+* and *pNf1f/f* mice, respectively (Figure 2D, multiple comparison Bonferroni’s post hoc test). Together with the distance and TAS data, these results suggest a delay in the acquisition of skills in *pNf1f/f* mice, or a moderate difficulty of *pNf1f/+* mice to achieve WT values during the first CW introduction. During the reintroduction to the CWs, the overall max speed achieved by *pNf1f/+* mice showed a modest difference vs. that of the WTs (Figure 2D right, two-way ANOVA; *p* < 0.029). Of note, no other parameter showed differences during CW reintroduction. Similar to the WTs, the comparison of N36 vs. N14 and N1 (Bonferroni’s post hoc tests, time as source of variability) suggested that the skills to reach the max speed that were learned during the first introduction are partially preserved after a 3-week break period (Supplementary Figure S1D). Regarding AAS (Figure 2E), both *pNf1f/+* and *pNf1f/f* mice showed lower speeds during the first CW introduction (two-way ANOVA; *p* = 0.005 and *p* = 0.0005, respectively) and different slope elevations compared with that of the WTs. A decrease in AAS was detected on N7–11 and N4–12 for *pNf1f/+* and *pNf1f/f* mice, respectively (multiple comparison Bonferroni’s post hoc test), suggesting a delayed development of skills to achieve WT values for AAS in female *pNf1* mutants.

During the first introduction to the CWs, the activity levels in both *pNf1* mutants were significantly lower (two-way ANOVA; *p* = 0.0074 for *pNf1f/+* and *p* < 0.0001 for *pNf1f/f*) and the slope was significantly different or showed a different elevation for hemizygous and homozygous *pNf1* mice (Figure 2F, inset). The nights with decreased activity were N10 for *pNf1f/+* and N1, N5–6, and N10 for *pNf1f/f* mice compared with WTs. Of note, the mean activity values showed considerable differences; however, the data dispersion precluded further statistical validation. No differences in activity levels were detected during the reintroduction to the CWs (N36–42). Similar to the WTs, *pNf1f/f* females showed higher activity levels in the second CW introduction (N1 vs. 36, Supplementary Figure S1H). Importantly, these data suggest that *pNf1* mutants are capable of running as well as the WTs after a break from the CWs, indicating a low potential for non-neurological factors (muscle, cardiac, energetic, or tumor issues) to influence CW performance. In summary, these findings suggest that both homozygous and hemizygous *Nf1* mutations in myelinating cells impact CW running performance, which delays or impairs learning but not memory in females; *pNf1f/f* mice eventually reach WT values for all CW parameters, while the values for *pNf1f/+* mice stay modestly below WT levels except for AAS. Max speed, AAS, and mainly activity level issues contribute to the abnormal curves for distance and TAS. Both *pNf1* mutants preserve CW running skills and activity, similar to the WTs, after a 3-week break.

### 3.4. Impaired Fine Motor Skill Acquisition following Myelin Nf1 Mutation in Male Mice

**Figure 3:**
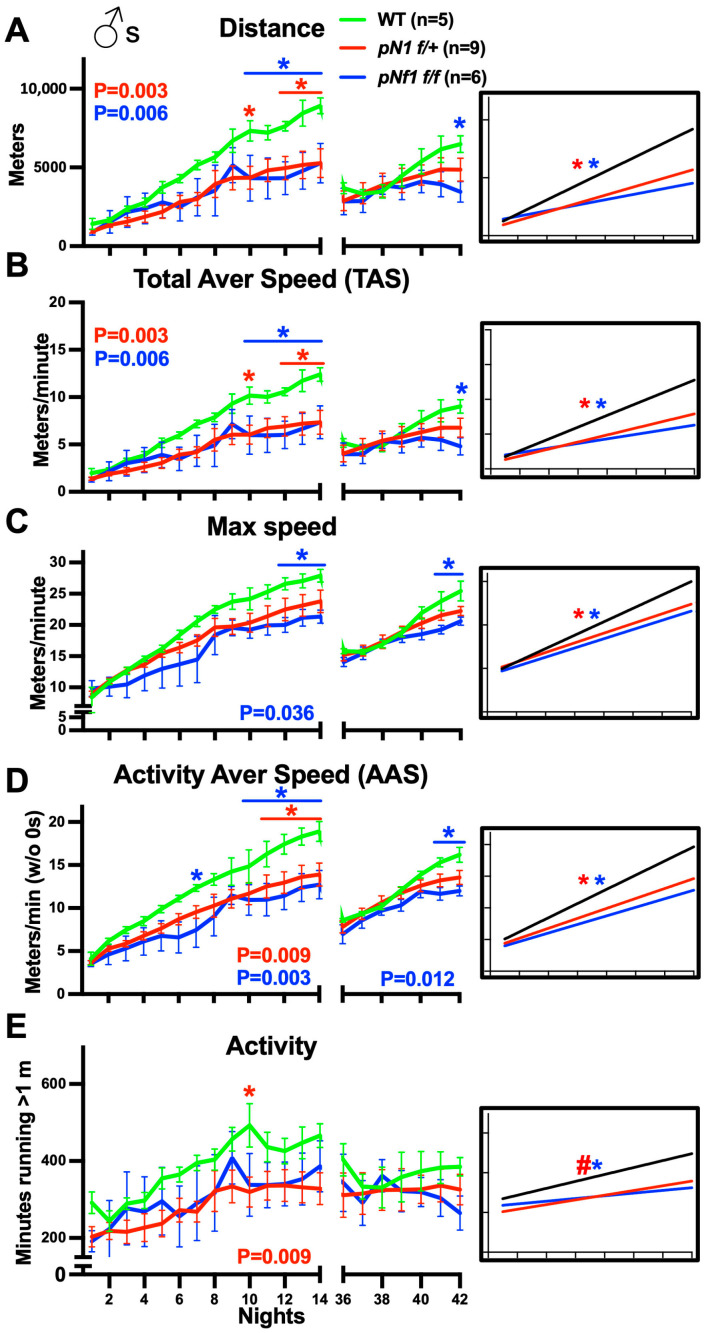
*pNf1* mutation in males impairs fine motor skill acquisition. X axis labels are shared in (**A**–**E**). (**A**–**E**) Nightly values for CW parameters for wild-type (green), *pNf1f/+* (red), and *pNf1f/f* (blue) mice are plotted: (**A**) total distance run, (**B**) Total Average Speed (TAS), (**C**) max speed, (**D**) Activity Average Speed (AAS), and (**E**) activity. Significant *p* values from two-way ANOVA tests (genotype as source of variation; WT n = 5; *pNf1f/+* n = 9; *pNf1f/f* n = 6) are shown under or above plots for the first (nights 1–14, left) or second (nights 36–42, right) introduction to CWs. Individual night comparisons for WT vs. *pNf1f/+* and WT vs. *pNf1f/f* (Bonferroni’s post hoc tests, * *p* < 0.05) are shown. Insets: comparison of slopes and intercept/elevation of slopes for WT vs. *pNf1f/+* and WT vs. *pNf1f/f* are shown (two-tailed linear regression, * *p* < 0.05 and # *p* < 0.05, respectively).

Male WT, *pNf1 f/f*, and *pNf1 f/+* mice were treated with tamoxifen at 2 MO and subjected to the CW test 2–4 and 2–6 months later. As shown in Figure 3A,B, the distance run and TAS per night in both *pNf1f/+* and *pNf1f/f* mice was significantly lower during the first introduction to the CWs (N1–14, two-way ANOVA; *p* = 0.003 for *pNf1f/+* and *p* = 0.006 for *pNf1f/f*, genotype as source of variation) and the regression analyses indicated significantly different slopes for nightly distance and TAS achieved (Figure 3A,B, inset) compared with WTs. In particular, significantly lower values were found on N10 and 12–14 for *pNf1f/+* and 10–14 for *pNf1f/f* male mutants (Figure 3A,B, multiple comparison Bonferroni’s post hoc tests). In contrast with female *pNf1* mutants, this indicates that two weeks of CW training is insufficient for *pNf1* males to develop skills to reach the peak values achieved by WT mice; the values increased similarly to the WTs’ from N1–5 but the rates show differences thereafter (Figure 3A,B). During reintroduction to the CWs (N36–42), the nightly distance and TAS achieved by both *pNf1* mutants was not different from those of the WTs (two-way ANOVA), except on N42 in *pNf1f/f* mutants (Figure 3A,B, right, Bonferroni’s post hoc test). In contrast with the WTs, no differences were found in distance on N1 vs. N36 in either of the *pNf1* mutants (Supplementary Figure S1C), further supporting the learning issues in male mutants, but precluding interpretations on skill memory issues. In contrast with females, the 14-night and 7-night periods of CW introduction and reintroduction might be insufficient for *pNf1* males to reach the WT peak values. Also, further differences among genotypes might require more time to develop in males, as suggested by the N42 values in *pNf1f/f* mice. Therefore, we conclude that at 2 weeks of training, male *pNf1* mutants show an impaired capability to achieve the same CW distance and TAS curves as WT mice.

The max speed achieved per night during the first introduction to the CWs showed modest but statistically significantly lower values only in *pNf1f/f* males (two-way ANOVA; *p* = 0.036, genotype as source of variability); nonetheless, the max speed slopes for both *pNf1* mutants were significantly different compared with that of the WTs (Figure 3C, inset, two-tailed simple linear regression). A decrease in max speed on N12–14 was found in *pNf1f/f* vs. WT mice (Bonferroni’s post hoc test). These data suggest that *Nf1* gene-dose dependent defects cause max speed differences during the first introduction to CWs. No overall differences were detected for either *pNf1* mutants during the reintroduction to the CWs (two-way ANOVA; *p* = 0.47 for *pNf1f/+* and *p* = 0.076 for *pNf1f/f*) and only *pNf1f/f* mice showed differences in the last 2 nights (Bonferroni’s post hoc test). Like in WT males, the max speed was higher on N36 in comparison with N1, but lower than N14 in *pNf1f/+* mutants; in *pNf1f/f*, the N36 value was not higher than the N1 value, further supporting the *Nf1* gene-dose-dependent defects in skill development (Supplementary Figure S1E).

Regarding AAS, significantly different learning curves (two-way ANOVA; *p* = 0.009 and *p* = 0.003) and slopes (Figure 3D inset, two-tailed simple linear regression) were detected for *pNf1f/+* and *pNf1f/f* mutants in the first CW introduction compared to the WTs. A decrease in AAS on N11–14 and N7, N10–14 were detected, respectively (Bonferroni’s post hoc test). These data indicate that *pNf1* mutants are unable to achieve a WT-like AAS. For the reintroduction to the CWs, only *pNf1f/f* mice showed significant differences (two-way ANOVA; *p* = 0.012, genotype as source of variability), mostly related to a decreased speed by the end of the period (N41–42). Similar to *pNf1* females, the activity levels in *pNf1* males showed high variability; yet, *pNf1f/+* males showed a significantly lower activity in the first CW introduction (two-way ANOVA; *p* = 0.009) and on N10 (Bonferroni’s post hoc test) and a different slope elevation (two-tailed simple linear regression), while *pNf1f/f* males only showed a significantly different slope (Figure 3E inset). For the reintroduction to the CWs, no changes in activity levels were found in either genotype, suggesting that *Nf1* male mutants present a similar drive/capability to run on CWs after a 3-week break. Overall, these data suggest that lower activity levels in male *pNf1* mutants contribute to changes in distance and TAS, yet to a lesser extent to changes in AAS (Figure 3D).

In summary, these results indicate that *Nf1* mutations in myelinating cells primarily impact the AAS and secondarily impact the activity levels in males, which contribute to an impaired ability of mutants to achieve the WT levels for distance and TAS in the CW. A modestly decreased max speed, mainly in homozygous *pNf1f/f* mice, could also contribute to the distance and TAS phenotypes. For the reintroduction period, *pNf1f/f* mice developed issues in their max speed and AAS, but not in their activity levels, which could affect the “re-learning” of skills at the end of the test. Finally, these results differ from those in *pNf1* females in the severity and timing of defects—skill development is delayed in females vs. impaired in males—and in the type of parameter contributing to the general phenotypes; all parameters were similarly impacted in females while mostly AAS was affected in *pNf1* mutant males.

### 3.5. Overall Similar Specificity and Fate of Recombined Cells in WT and pNf1 Mice of Both Genders

**Figure 4:**
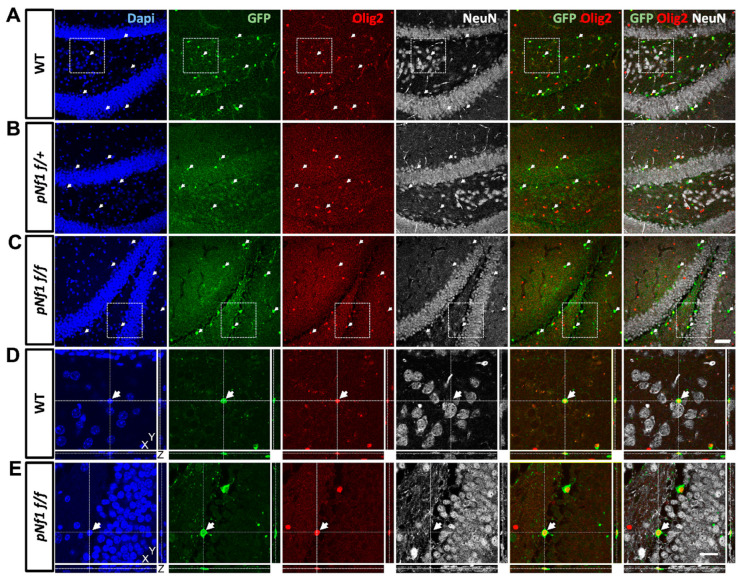
Recombination driven by *PlpCre^ER^* is highly specific for OL lineage cells in the adult hippocampus. Representative images of brain sections containing the hippocampus of WT (**A**,**D**), *pNf1f/+* (**B**), and *pNf1f/f* (**C**,**E**) mice subjected to CW tests. Sections were immunostained to detect the reporter EGFP (green), the OL-lineage marker Olig2 (red), and the neuronal marker NeuN (white). Cell nuclei are labeled with DAPI (blue). Arrows indicate EGFP+ recombinant cells that are also Olig2+ but not NeuN+. (**D**,**E**) High magnification orthogonal projection (Z axis indicated at bottom right) of areas in (**A**,**C**) (dotted line) depicting EGFP+Olig2+ cells in close proximity to, but independently from, NeuN+ cells. (**A**–**C**) scale bar = 50 μm, (**D**,**E**) scale bar = 20 μm.

Plp1 is the most abundant protein in CNS myelin [62] and the *Plp/Cre* allele is widely used to target recombination to OLs and to a lesser extent, to Schwann cells [50]. Nonetheless, *PlpCre* recombines outside the myelinating lineage, mostly during embryonic–postnatal development [63,64]. Using the inducible *PlpCre^ERT2^* allele in adults improves myelinating-cell specificity, with no recombination in astrocytes, endothelial cells, microglia, or OPCs but potentially scattered recombination in hippocampal neurons [45,50]. Hence, to evaluate the potential neuronal induction of the *Nf1* mutation *in pNf1* mice, we immunostained thick (35 μm) brain sections of WT, *pNf1f/+*, and *pNf1f/f* mice using the neuronal marker NeuN and the recombination reporter ccEGFP. No obvious NeuN-EGFP co-localization signals were observed in the hippocampus (Figure 4), motor cortex, corpus callosum (CC; Supplementary Figure S4), or basal ganglia (striatum; Supplementary Figure S5) in any of the genotypes. Although the 3D confocal analysis demonstrated a close proximity of EGFP+ with NeuN+ cells, the EGFP signals were only co-localized with the OL lineage marker Olig2 in WT and *pNf1* mutants (Figure 4, Supplementary Figure S4 bottom). Therefore, a negligible contribution of *Nf1* mutant neurons to the CW phenotypes is suggested.

**Figure 5:**
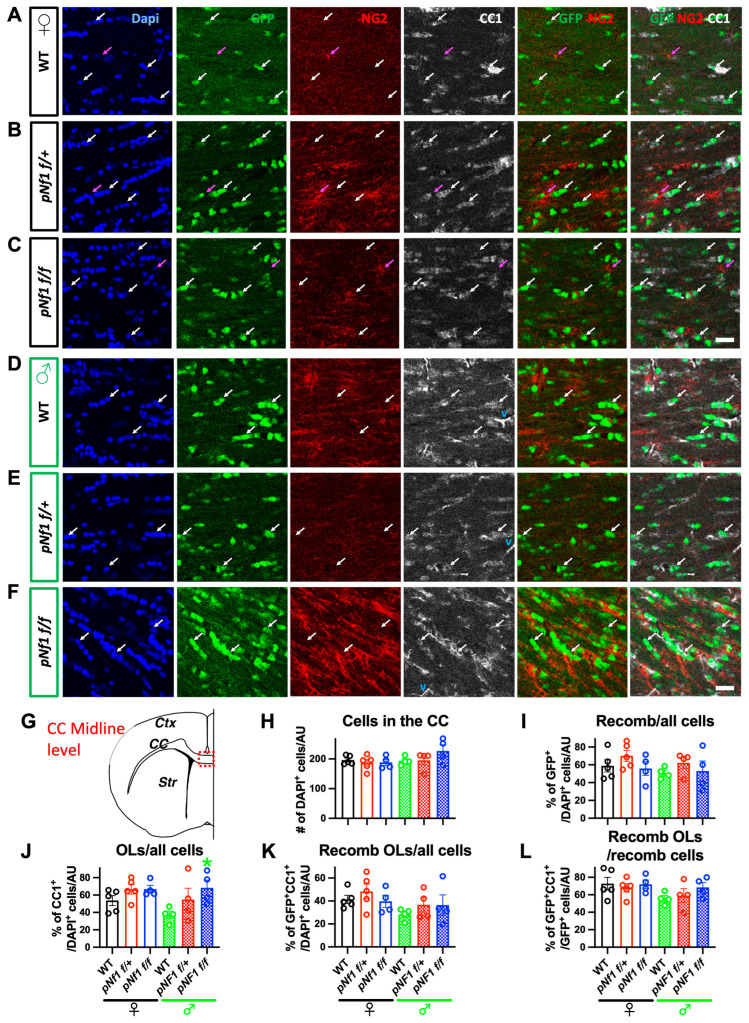
Number/fate of recombined cells is comparable in the CC of WT and *pNf1* mice subjected to CW tests. X axis labels shared in H, K, and I, L. Representative images from female (**A**–**C**) and male (**D**–**F**) brain sections containing the CC ((**G**), midline region) of WT (**A**,**D**), *pNf1f/+* (**B**,**E**), and *pNf1f/f* (**C**,**F**) mice subjected to CW tests. Sections were immunostained to detect the reporter EGFP (green), the oligodendrocyte progenitor (OPC) marker NG2 (red), and the OL marker CC1 (white). Cell nuclei are labeled with DAPI (blue) and non-specific CC1 signals in some blood vessels are indicated with a “v” (cyan). Pink arrows show NG2+EGFP- non-recombinant OPCs in close interaction with OLs. White arrows indicate EGFP+ recombinant cells co-localizing with CC1 but not with NG2. Scale bar = 25 μm. Cell quantification of all DAPI+ cells (**H**), % of EGFP+ recombinant cells/DAPI+ cells (**I**), CC1+ OLs/DAPI+ cells (**J**), EGFP+CC1+ recombinant OLs/DAPI+ cells (**K**), and EGFP+CC1+ recombinant OLs/recombinant cells (**L**). Student’s *t* test: * *p* < 0.05 (n = 4 mice/genotype/gender). Data are shown as the mean + SEM.

Shortly after recombination in *pNf1f/f* and *p*WT mice, ~75% of the cells marked by a reporter gene were CC1+ OLs in the CC and ~35% of the CC1+ OLs were reporter gene positive in the optic nerve [45]; the recombinant cell density is similar in WT and *pNf1f/f* mice for up to 6 months [44]. However, gender-dependent recombination efficiency/specificity and *Nf1* mutation-driven changes in the fate of recombinant cells has not been explored. Therefore, using immunofluorescence, we assessed the distribution and identity of recombinant and non-recombinant OL lineage cells in the CC of WT and *pNf1* mice subjected to the CW test. The densities of all DAPI+ and EGFP+ recombinant cells were comparable in the CC (midline level) of female and male *pNf1f/+* and *pNf1f/f* mice vs. WTs (Figure 5A–I, unpaired *t*-test, * *p*< 0.05). A modest increased percentage of EGFP+ recombinant cells among all DAPI+ cells was observed at the cingulum level of the CC of *pNf1f/+* females (Supplementary Figure S6I), suggesting gender-specific regional differences in recombination efficiency and/or the increased survival of recombinant cells. Interestingly, *pNf1f/f* males (but not females) showed a higher percentage of CC1+ OLs among all cells in both CC regions analyzed (Figure 5J and Supplementary Figure S6J); nonetheless, the density of recombined OLs did not show differences (Figure 5K,L, and Supplementary Figure S6K,L), indicating a non-cell-autonomous origin of this phenotype. Similarly, an increased number of non-recombinant OPCs (NG2+EGFP-) was observed at the CC midline level of *pNf1f/+* females (Supplementary Figure S7B). In summary, the recombination driven by *PlpCre^ER^* targets OLs with high specificity and similarly in males and females. Modest regional variations in the fate of recombined cells in *pNf1f/+* females and broader non-cell-autonomous effects in the OL lineage of *pNf1* males suggests that gender-specific responses to the *Nf1* mutation contribute to the motor skill learning deficits (Figure 2 and Figure 3); yet, these are secondary to the strong intrinsic gender-driven CW learning differences (Figure 1).

### 3.6. Single-Night Analyses Suggest Nf1 Gene Dose- and Gender-Specific Defects in CW Performance

**Figure 6:**
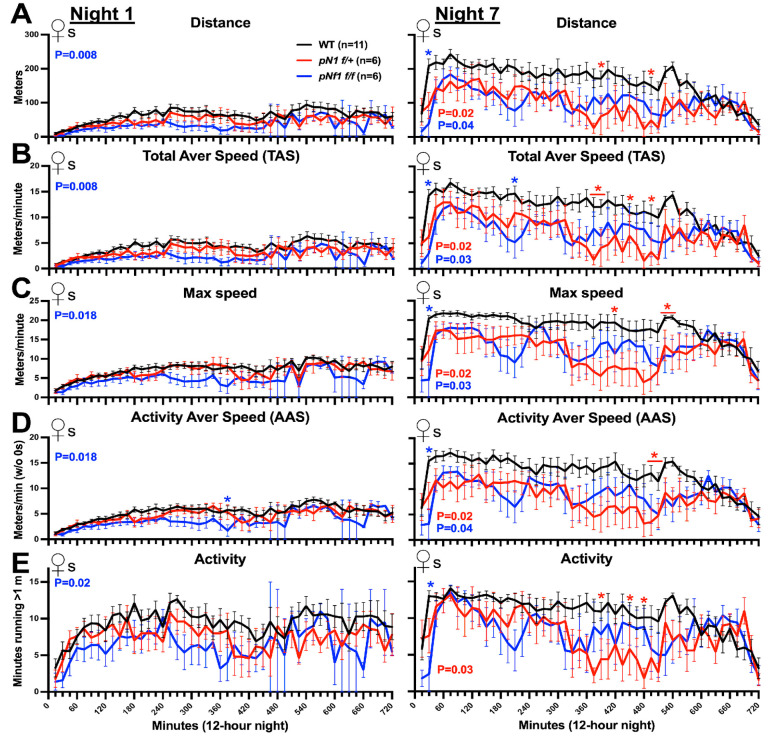
Progressive and gene-dose-dependent CW running issues from N1 and N7 in *pNf1* mutant females. X axis labels are shared in (**A**–**E**). (**A**–**E**) Data from night 1 (N1, left) and night 7 (N7, right) of the first introduction to CWs were divided into 15 min intervals and CW parameters for wild-type (black), *pNf1f/+* (red), and *pNf1f/f* (blue) mice were plotted: (**A**) total distance run, (**B**) Total Average Speed (TAS), (**C**) max speed, (**D**) Activity Average Speed (AAS), and (**E**) activity. Significant *p* values from two-way ANOVA tests with genotype as the source of variation are shown for each plot. Individual night comparisons for WT vs. *pNf1f/+* and WT vs. *pNf1f/f* (Bonferroni’s post hoc tests, * *p* < 0.05) are shown.

Immediate (in hours) learning defects in *pNf1* mice might impact CW performance as the introduction of WT mice to CWs causes OL precursors to quickly differentiate in ~4 h [36,57] and the basal differentiation rate of OLs is increased in *pNf1f/f* mice (non-cell-autonomous defect) [44]. Whether hourly CW performance abnormalities are present in *pNf1* mutants, but masked in the whole-night analyses, is unknown; therefore, we analyzed the CW data by separating N1 in intervals of 15 min. As shown in Figure 6A,B left, female *pNf1f/f* but not *pNf1f/+* mice showed significant differences in distance and TAS on N1 as a whole compared to the WTs (two-way ANOVA; *p* = 0.0088 and *p* = 0.19, respectively, genotype as source of variation); no specific hours showed significant differences (Bonferroni’s post hoc test). Similarly, *pNf1f/f* (but not *pNf1f/+*) females showed different curves for max speed, AAS, and activity (two-way ANOVA; *p* = 0.018, *p* = 0.018, and *p* = 0.02). This suggests that there are early (N1) *Nf1* gene-dose effects in females. The analysis of N1 in 15 min intervals in male *pNf1* mutants did not show statistically significant differences in any CW parameter (Supplementary Figure S8A–E left, two-way ANOVA; genotype as source of variation); in fact, the curves consistently overlapped among genotypes, including in the periods of highest and lowest activity (Supplementary Figure S8E, left). Overall, *pNf1f/+* mutants of both genders and *pNf1f/f* males did not show hourly running defects on N1; however, *pNf1f/f* females show early learning issues, potentially related to non-cell-autonomous issues affecting OL differentiation.

To determine whether the phenotypes found in the nightly analyses (Figure 2 and Figure 3) originate from specific periods, we analyzed 15 min intervals from N7 in both females and males. Significant differences (Figure 6A–E, two-way ANOVA, genotype as source of variation vs. WT) for distance, TAS, max speed, and AAS were detected in female *Nf1f/+* (*p* = 0.019, *p* = 0.017, *p* = 0.015, and *p* = 0.02, respectively) and *pNf1f/f* (*p* = 0.037, *p* = 0.027, *p* = 0.029, and *p* = 0.037, respectively) mice. The number of minutes with activity significantly decreased in *pNf1f/+* but not in *pNf1f/f* females (*p* = 0.029 and *p* = 0.053). Intervals with decreased values (Bonferroni’s post hoc test) shown by *pNf1f/+* females were distributed in the second half of the night; the activity levels overlapped with that of the WTs up to minute 300, followed by a decrease lasting ~4 h, suggesting abnormalities in the factors regulating running motivation or endurance. Thereafter (last 2 h), *pNf1* and WT females showed an overlapping decrease in all CW parameters (Figure 6, right), suggesting unaffected night activity.

By N7, *pNf1f/+* male mice showed significant differences for all CW parameters (Supplementary Figure S8, two-way ANOVA; distance, *p* = 0.01; TAS, *p* = 0.004; max speed, *p* = 0.04; AAS, *p* = 0.04; and activity, *p* = 0.007; genotype as source of variation) and *pNf1f/f* only showed differences for max speed (*p* = 0.022). Interestingly, males and females showed different N7 running schedules; for example, WT mice of both genders achieve peak values for all CW parameters shortly after the “night” started, but these values in males gradually decreased thereafter, while the females maintained the peak values for most of the night (compare distance and TAS for N7 in Figure 6A,B vs. Supplementary Figure S8A,B); the hourly activity could be a main determinant for gender-dependent performance in the CW. In summary, *pNf1* phenotypes are influenced by *Nf1* gene-dose effects during N1 in females and N7 in males. By N7, *pNf1* female phenotypes are influenced by changes during the second half of the night while male phenotypes are not influenced by changes during specific periods.

### 3.7. Regulation of Nitric Oxide Production Differentially Impacts Learning in Female and Male pNf1 Mice

**Figure 7:**
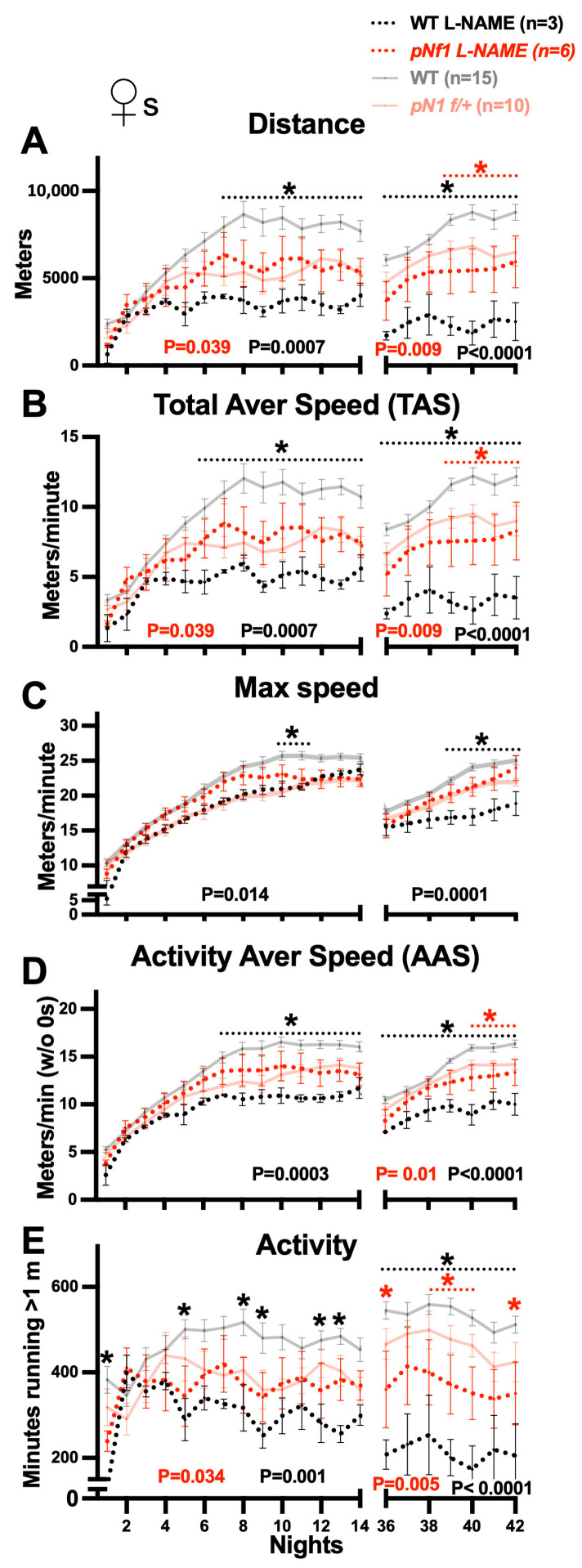
L-NAME treatment modifies learning curves in *pNf1* and WT females. X axis labels are shared in (**A**–**E**). Plots for nightly values of CW parameters for WT (black dotted line) and *pNf1 f/+* (red dotted line) female mice treated with L-NAME (0.3 mg/L in drinking water): (**A**) total distance (meters), (**B**) Total Average Speed (TAS), (**C**) max speed, (**D**) Activity Average Speed (AAS), and (**E**) minutes with activity. Plots for untreated WT and *pNf1* females are shown in faded colors as control values (compare with Figure 2). Statistically significant *p* values (two-way ANOVA test, treatment as source of variation) are shown under the plots for first (left) and second (right) introduction to CWs (* *p* red: L-NAME-treated *pNf1 f/+* vs. untreated WT; * *p* black: L-NAME-treated WTs vs. untreated WTs). No differences between untreated vs. L-NAME-treated *pNf1 f/+* mice were found. Comparison of individual nights are also shown (same color code, Bonferroni’s post hoc tests, * *p* < 0.05).

Brief daily treatment with the NO synthase 1–3 (NOS1–3) inhibitor N-nitroarginine methyl ester (L-NAME) rescues the phenotypes found 5–6 months post-tamoxifen treatment in *pNf1 f/+* mice: abnormal myelin decompaction [44], reduced motor coordination, and MRI abnormalities including CC microstructure (fractional anisotropy) and interhemispheric functional connectivity abnormalities [48]. To test the regulation of fine motor skill learning by NO signaling, we subjected *pNf1 f/+* and WT mice to oral treatment with L-NAME (0.3 g/L in drinking water) 4 days before and during the CW test. Specific responses were found in female/male and WT/mutant mice (Figure 7A–E); the learning curves for distance (*p* = 0.039), TAS (*p* = 0.039), and activity (*p* = 0.034) remained significantly lower in L-NAME-treated *pNf1 f/+* females compared with untreated WTs, but the curves for max speed (*p* = 0.059) and AAS (*p* = 0.14) did not showed differences compared to those of the WTs (two-way ANOVA, treatment as source of variation; WT n = 15 (6 added to those in Figure 2) and *pNf1* n = 6), suggesting a rescue of these two specific phenotypes. Nonetheless, all CW parameters in treated *pNf1f/+* females showed no significant differences compared with those of untreated *pNf1 f/+* females. Interestingly, despite initially comparable values from N1 to ~N7, all the learning curves for L-NAME-treated WT females were severely affected during both the first and second CW introduction compared with untreated WTs (distance and TAS, *p* = 0.0007 and *p* < 0.0001; max speed, *p* = 0.014 and *p* = 0.0001; AAS, *p* = 0.0003 and *p* = 0.0001; and activity, *p* = 0.001 and *p* < 0.0001). These results suggest a strong influence of NO signaling on the normal development of fine motor skills; however, if NO is abnormally increased in *pNf1* mice—as previously suggested [44]—NOS inhibition could revert the CW learning phenotypes. Considering the gender-dependent learning phenotypes, we subjected L-NAME-treated *pNf1 f/+* males to the CW test 5–6 months after *Nf1* mutation induction. In contrast to its effect on *pNf1* females, L-NAME had a negative impact on the learning curves of *pNf1* males compared with untreated WTs (Supplementary Figure S9; two-way ANOVA, treatment as source of variation; WT n = 19 (10 added to those in Figure 2) and *pNf1* n = 6; distance and TAS, *p* < 0.0001; max speed, *p* = 0.004; AAS, *p* = 0.001; and activity, *p* ≤ 0.0001). Furthermore, a comparison of L-NAME-treated vs. untreated *pNf1* males indicated a lower development of motor skills (Supplementary Figure S9; two-way ANOVA, treatment as source of variation; distance and TAS, *p* < 0.004; max speed, *p* = 0.003; AAS, *p* = 0.009; and activity, *p* ≤ 0.004), also contrasting with the effects on females. This supports the idea that the *Nf1* mutation impacts NO signaling and suggests that the parameters influencing the learning curves of fine motor skills in *pNf1* males are highly sensitive to NO changes.

## 4. Discussion

Studying a monogenic disease such as NF1 represents an opportunity to understand the mechanisms of brain physiology and pathophysiology. In particular, NF1 research has great potential to unveil brain dysfunction related to abnormal white matter and myelin. However, since the description of WM abnormalities in NF1 [32], the idea of abnormal WM/myelin driving NF1 neurological issues remains controversial. Patient abnormalities include defects in myelination timing/quality, myelin turnover, vacuolation or decompaction, and demyelination [65,66,67], but no experimental data can prove or refute abnormal myelin–behavior links. Here, we adapted the CW test, used to show the impact of defective myelination on the learning of fine motor skills, to study the impact of an *Nf1* mutation in pre-existing myelinating cells on learning and memory. *Nf1* mutant females showed delayed or impaired acquisition of CW motor skills, while males mostly showed an inability to achieve WT-level skills. Overall, the memory of acquired skills was less impacted by *Nf1* mutations. The gene dose of the *Nf1* mutation modestly modified the progression of phenotypes, mostly in females, and a treatment to decrease NO showed modest positive effects in learning for females, but negative effects in males. A subtle regional variability in the fate of recombinant/mutant OLs and non-cell-autonomous abnormalities were detected in *pNf1* mutants, but these did not correlate with any specific learning issues. Overall, our results are the first insights for a specific mutation of *Nf1* in mature myelinating cells impacting learning of fine motor skills.

### 4.1. The CW Running Test Is a Robust Assessment for NF1 Learning/Memory Issues Regulated by Central Myelin in Mice

Learning changes the brain WM–myelin structure in humans and rodents [36,57,68,69,70,71,72,73,74,75] and abnormal myelin underlies or contributes to cognitive issues in disease [76,77,78]. Diverse tests [79,80,81,82,83], including voluntary wheel running [36,57,84,85], have been used to link myelin plasticity with normal and defective learning. In particular, the CW test was used to show that learning increases myelination in the corpus callosum (CC) and blocking myelination precludes mastering the CW [36]. Hence, we hypothesized that an *Nf1* mutation in myelinating cells will impact the learning/memory of CW fine motor skills. This test was selected since its unbiased/automated recordings deliver raw data for the analyses of diverse parameters with minute to weekly resolution and using fewer mice [36,57] compared with common learning tests. Yet, a main experimental challenge to studying myelin–behavior links in NF1 has been separating central myelin-dependent mechanisms from neuronal or other compromised systems. Indeed, the *PlpCre* system might induce recombination in hippocampal neurons [45,50]; however, our recombination analyses support negligible number of neurons with *Nf1* mutation in the cortex, hippocampus, and basal ganglia (striatum). This high specificity is likely related to the induction of recombination in adult mice. Moreover, while spatial learning issues in *Nf1^+/−^* mice have been associated with the hippocampus [49], CW learning seems less dependent on the hippocampal machinery [86]. Demyelination does not affect performance in regular wheels (RWs) but it does affect CW performance [84], suggesting the involvement of additional motor control circuits, which are likely needed for asymmetrical gait/bilateral coordination [36,57,84]. Lastly, sleep disorders may affect NF1 patients [54,60,61]; while we did not observe changes in voluntary wheel activity (a mostly nocturnal natural behavior [87]), the actual circadian rhythm challenges can be added to further NF1 research using the CW test.

### 4.2. Separating Motor Learning from Motor Competency Issues

A common feature in NF1 patients is the development of peripheral tumors. In fact, homozygous *pNf1* mutations in adult mice cause neurofibroma formation (grade I-like) that requires them to be sacrificed 5 to 13 months post-tamoxifen treatment [55]. Therefore, our protocol excluded mice of this genotype and period that could impact the capability of mice to master the CW. Overall, the direct impact of abnormal central myelin on CW fine motor learning is supported by many pieces of evidence. (1) It was established that tumor formation in NF1 requires *Nf1* loss of heterozygosity, but no evidence suggests its requirement for neurological issues; consistent with this, hemizygous *pNf1* females show no tumors [55] but displayed robust CW learning curve issues. (2) No overall signs of disease or distress were observed at any point during the CW test; body weight and general health was longitudinally assessed during the experiments and necropsies detected only one mouse with a peripheral tumor and that mouse was removed from the study. (3) WT and *pNf1* mutants started the CW tests with similar basal speeds/activity levels (N1), but these values increases at different rates in response to genotype and gender. Moreover, after a 3-week break, the initial CW values were again similar to those of the WTs, but higher than N1 values and lower than N14 values. (4) Homozygous *pNf1* females (that potentially develop nerve tumors 5–13 months post-tamoxifen treatment [55]) achieved the same max speed as WTs in the first CW introduction, and the max speed values were similar in all genotypes/genders during the CW reintroduction. This suggests that *pNf1* mutations do not significantly affect the elements controlling max running speed, including the muscles, bones, and heart. Indeed, it was proposed that the CW test is reliable in revealing central motor deficits during de/remyelination, minimizing the contribution of cardiopulmonary and musculoskeletal training [88]. Still, future mechanistic research should include assessments of energy metabolism and motivational issues.

### 4.3. Phenotypes of pNF1 Mice in the Context of Intrinsic Gender-Related Learning Differences

No gender-related differences in CW performance were previously detected [36,57]; however, we observed that females outperformed males, regardless of WT or *Nf1* mutant genotype, in all CW parameters. We attribute these apparently conflicting results to variations in the strain genetic background, as suggested by gender differences in regular wheel (RW) performance in specific mouse strains [58,59]. Importantly, the genetic background and number of backcrossing generations of genetically modified mice are highly heterogeneous among research groups, and therefore represent sources of variation. In addition to robust WT gender-intrinsic differences, changes driven by *pNf1* mutations were detected; a delay in achieving the peak distance and TAS—influenced by decreased max speed, AAS, and potentially activity levels—was found in *pNf1f/f* females, while *pNf1f/+* females were not able to achieve the WT values for distance and TAS. Homozygous and hemizygous *pNf1* males did not achieve the WT values for distance and TAS, which was mostly influenced by issues with AAS followed by activity levels, and a minor contribution of a decreased max speed. Whether neurofibromin regulates sex hormone biology during brain development and/or adult stages is an important question to explore, particularly considering that NF1 patients and models show gender-dependent neurological issues [1,6,52,89,90]. For example, depression symptoms are more common in women with NF1 [91] and they have an increased risk of unipolar depression and ADD [15,92]. As CW running is a voluntary activity associated with motivation [36], whether this test can inform about gender-related mechanisms of depression and ADD—a frequent issue in NF1 [93]—is an intriguing possibility.

### 4.4. Impact of L-NAME Treatment on CW Learning of pNf1/WT Male/Female Mice

The NO pathway is hyperactive in *pNf1* mice and NO synthesis inhibition rescues various phenotypes [44,48]; it was hypothesized that short-term treatment with L-NAME quickly re-establishes normal NO levels in the brain and rescues myelin structure and brain connectivity. In this study, the impact of L-NAME treatment on CW learning of *pNf1* females was inconclusive; the max speed and AAS were rescued compared with untreated WTs, but the learning curves were similar to those of untreated *pNf1* females. Interestingly, a clear/strong impact of L-NAME treatment on CW learning curves in WT females was detected, when compared with untreated ones; thus, the influence of NO signaling on CW learning is supported. Moreover, L-NAME elicited a strong negative response on learning in male *pNf1* mutants. Although all NOS isoforms seemed to be increased in *pNf1* mice regardless of the gender [45] (which justified the pan-NOS inhibition in our study), independently testing inhibitors for specific isoforms in both genders, particularly those evaluated in humans [94], hold the potential of rescuing learning issues in *pNf1* mice and patients. We speculate that NOS isoforms expressed in various brain cell types contribute to homeostatic/balanced NO signaling in WT mice; L-NAME would decrease NO to levels affecting learning. On the other hand, the abnormally increased NO signaling caused by the *Nf1* mutation would be reduced by L-NAME to near-normal levels in *pNf1* females; however, in *pNf1* males, L-NAME would reduce NO levels as it does in WTs, negatively impacting learning. Research on specific NOS isoforms in OLs and their control will shed light onto the specific mechanisms in males/female *pNf1* mice.

### 4.5. Potential Central Myelin Mechanisms for the Impact of Nf1 Mutation on Learning

**Figure 8:**
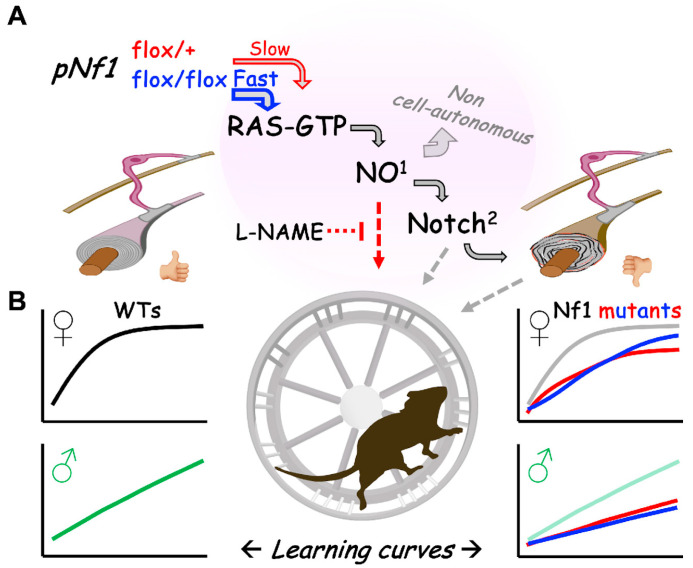
Working hypothesis for CW motor learning issues following *Nf1* mutation induction in myelinating cells. (**A**) Hemizygous (red) or homozygous (blue) *Nf1* mutation in myelinating cells (*pNf1* mice) increases RAS/MAPK, nitric oxide (NO [45])^1^, and Notch [44]^2^ signaling ~6 months (slow) or ~1 month (fast) post-mutation induction. Myelin decompaction (right) and non-cell-autonomous (top) defects are downstream effects. (**B**) Learning curves of fine motor skills (voluntary CW running) show gender-dependent differences in WT mice (left). Additionally, an *Nf1* mutation in myelin affects learning curves in both female and male *pNf1* mice, with a modest influence of the *Nf1* gene dose (right, red vs. blue lines). Defective NO signaling regulates CW learning in WT and *pNf1* mice by unknown mediators (red dotted arrow). Abnormal downstream Notch signaling and myelin decompaction (gray dotted arrows) might contribute to motor learning issues.

How could an *Nf1* mutation in pre-existing oligodendrocytes impair learning? At 6–9 months post-*Nf1* mutation induction, hemizygous *pNf1* mice present myelin decompaction [44] and reduced dtiMRI fractional anisotropy in the CC, in parallel with reduced interhemispheric functional connectivity of the motor cortex; these defects are consistent with disrupted central myelin integrity [48]. In parallel, deficits in sensory gating in the pre-pulse inhibition of the startle response [44] and in gross rota rod motor coordination were described [48]. These phenotypes developed in *pNf1* hemizygotes, indicating that there is no need for a “second hit” mutation. Learning phenotypes did not require *Nf1* mutation homozygosity in our study either, but the mutation dose modulated the timing and severity of phenotypes. Nonetheless, it was reported that the *pNf1* mutation dose controls the time to development of myelin decompaction and the sensitivity to phenotype-rescuing drugs: (1) *pNf1* f/f mice reach ~65% decompacted myelin fibers in ~1 month, while *pNf1* f/+ mice reach this level in >4 months; neither genotype further increases this percentage of decompacted fibers. (2) Decompaction is rescued by single drugs targeting MAPK, NO, or Notch signaling in *pNf1* f/+ mice but not in *pNf1* f/f mice [44]. This line of evidence builds a scenario in which the normal expression level of *Nf1* maintains the myelin structure (including but not limited to compaction), OL signaling, and brain connectivity required to respond to learning challenges such as the CW (Figure 8). In addition to the impact of OL *Nf1* mutations on mature myelin, non-cell autonomous effects ([44] and here) can impact the formation of new myelin. However, the lack of phenotypes in *pNf1 f/+* mutants in the first hours/days in the CW test (period for OL differentiation and progenitor proliferation [57]) suggests a lower degree of influence of this mechanism. Instead, we propose that in *pNf1* mice subjected to the CW, defective myelin formed or remodeled by pre-existing *Nf1* mutant OLs [95,96,97,98] causes a defective response to the learning challenge (Figure 8). Abnormal signaling in the *pNf1* tissue environment [44]—for example, through NO—could produce non-cell-autonomous responses and further defects, for example, in the structure of newly formed WT myelin.

Discovering the detailed mechanisms driving the learning defects described here will require focused research aimed at identifying gender-dependent and independent factors, for instance, those driving CW learning differences in WT mice (the most prominent) that are independent of *Nf1* mutations. These gender-dependent factors potentially involve different biological processes of OPCs and myelin in males and females, including differential basal OPC density/survival and responses to myelinating challenges [99,100,101,102]. Moreover, NO modulation of CW learning is inferred from the detrimental response of WT mice to L-NAME, together with its marginal (in females) to robust (in males) impact in *pNf1* mouse learning. A detailed pharmacological screening of NOS-specific inhibitors, with an emphasis on those that have been tested in humans [94], will shed light on more specific mechanisms and could unveil therapeutic tools for clinical use in NF1 neuropathology.

## 5. Conclusions

We propose that neurofibromin expression in myelin-producing adult OLs is required for proper learning. The neurological issues in adult NF1 patients, including learning problems, depression, and dementia, are influenced by gender and *Nf1* mutations could participate in the complexity of their pathophysiology. Due to their lifelong and brain wide impact, myelin defects could be the main contributors to NF1 neuropathophysiology. This preclinical study shows that a highly specific *Nf1* mutation in adult myelinating cells impacts the timely and accurate learning of fine motor skills. If similar mechanisms exist in patients, our myelin-focused model will contribute to the development of preventive/therapeutic interventions based on restoring or enhancing myelin function, using gender-tailored and time-specific schedules. Drugs that improve myelin function, including those that are currently in clinical trials for demyelinating diseases, could be repurposed to treat NF1 using schedules derived from fundamental research.

## Data Availability

Requests to access the datasets should be directed to alejandro.lopezjuarez1@utrgv.edu.

## Supplementary Materials

The following supporting information can be downloaded at: https://www.mdpi.com/article/10.3390/cancers16030477/s1, Figure S1: Memory of acquired fine motor skills is not disrupted by *Nf1* mutation in myelinating cells; Figure S2: No overall impact of *pNf1* mutation on body weight; Figure S3: No overall impact of *pNf1* mutation on nocturnal running activity in the CW test; Figure S4: Recombination driven by *PlpCre^ER^* is highly specific for OL lineage cells in the motor cortex and CC; Figure S5: Recombination driven by *PlpCre^ER^* is highly specific for OL lineage cells in the Striatum; Figure S6: Density and fate of recombined cells is comparable in the CC of WT and *pNf1* mice; Figure S7: Non-cell-autonomous response of OPCs in the CC of *pNf1* mice; Figure S8: Progressive CW running issues from N1 to N7 in *pNf1* mutant males; Figure S9: L-NAME treatment negatively impacts learning curves in *pNf1* males.
