# Supplementary material for: Gender-Specific Fine Motor Skill Learning Is Impaired by Myelin-Targeted Neurofibromatosis Type 1 Gene Mutation
Source: Cancers (Basel). 2024 Jan 23;16(3):477. doi: 10.3390/cancers16030477 (PMC10854893; doi:10.3390/cancers16030477)
Supplement: Supplementary file 1 [file cancers-16-00477-s001.zip › cancers-2833337-supplementary.pdf]

cancers MDPI

Gender-Specific Fine Motor Skill Learning Is Impaired by Myelin-Targeted Neurofibromatosis Type 1 Gene Mutation

Daniella P. Hernandez [1,†], Daniela M. Cruz [1,†], Celeste S. Martinez [1], Larisa M. Garcia [1], Ashley Figueroa [1], Marisol Villarreal [1], Liya M. Manoj [1], Saul Lopez [1], Karla D. López-Lorenzo [2] and Alejandro López-Juárez [1,*]

[1] Department of Health and Biomedical Sciences, University of Texas Rio Grande Valley, Brownsville, TX 78520, USA
[2] Department of Biology, Texas A&M University, College Station, TX 77843, USA
* Correspondence: alejandro.lopezjuarez1@utrgv.edu
† These authors contributed equally to this work.

SUPPLEMENTARY INFORMATION: 9 supplementary figures + figure legends

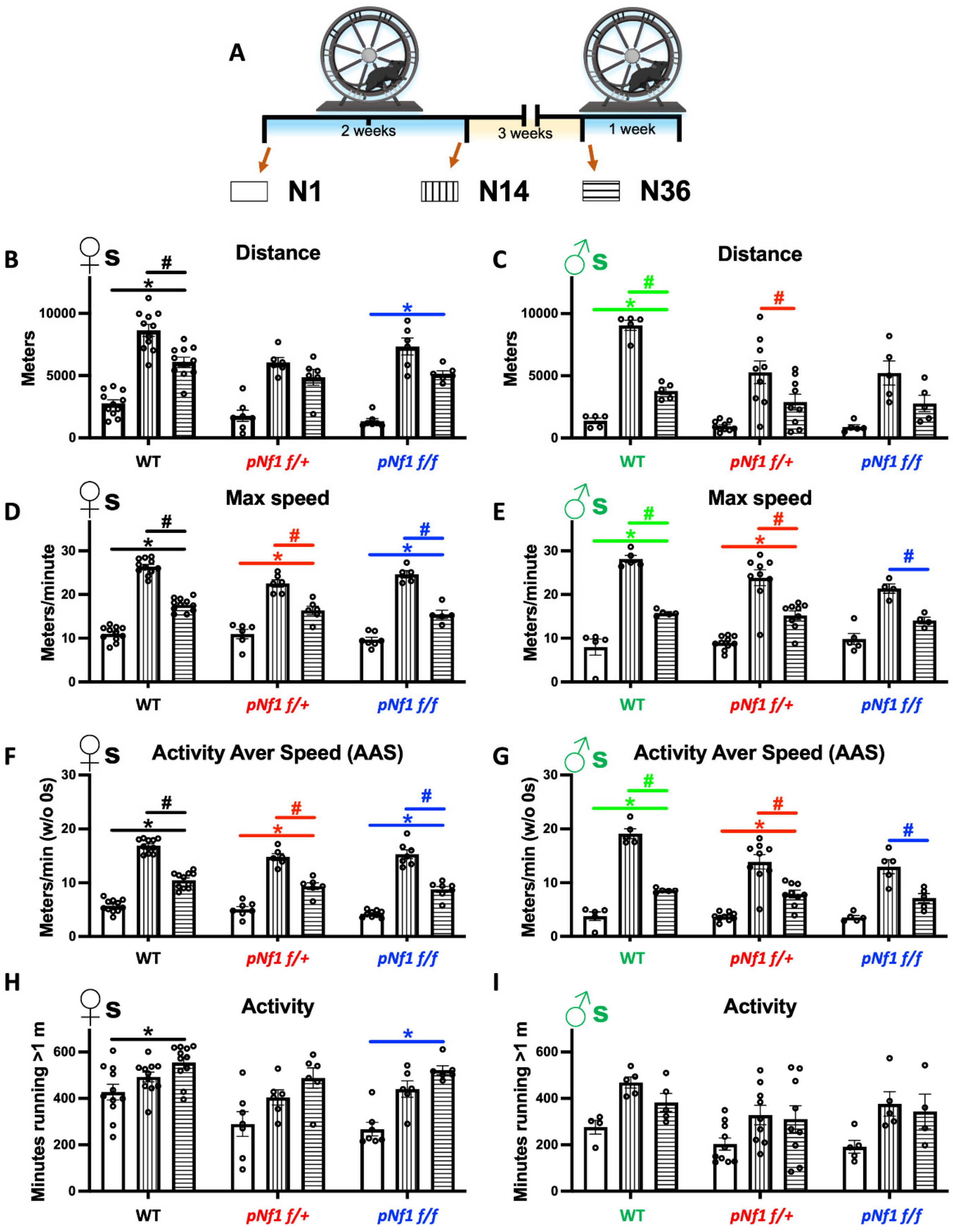

Supplementary Figure S1**. Memory of acquired fine motor skills is not disrupted by *Nf1* mutation in myelinating cells**. A) CW learning/memory protocol depicting night 1 (N1; first introduction of mice to CWs, empty box), N14 (end of first CW introduction and plateau of skills; box with perpendicular lines), and N36 (beginning of second introduction to CWs after a 3-week break; box with horizontal lines). B-I) Statistical analysis for distance (B-C), Max speed (D-E), Activity Average Speed (AAS; F-G), and activity (H-I); two-way

ANOVA with time as source of variation within every genotype/gender. Comparisons for N1 *vs.* N36 (initial value in first *vs.* second introduction, *p<0.05), and N14 *vs.* N36 (end of first introduction *vs*. initial value of second introduction, #p<0.05), for female (B,D, F, and H) and male (C, E, G, and I), WT (black, green), *pNf1f/+* (red), and *pNf1f/f* (blue) mice are shown (Bonferroni's post-hoc tests; WT females n=11, males n=5; *pNf1f/+* females n=6, males n=9; and, *pNf1f/f* females n=6, males n=6). **Notes**: both female and male WT mice show decreased values for distance, max speed, and AAS in N36 as compared to N14, but N36 values are still higher as compared with N1, suggesting partial permanence of CW running skills after a 3-week break. Male/female *pNf1f/+* mice and female *pNf1f/f* show the same pattern for Max speed and AAS, supporting normal permanence of CW running skills. Distance values in male/female *pNf1f/+* and male *pNf1f/f* mice do not show differences between N1 and 36, likely reflecting the previously observed learning (not memory) issues. Female *pNf1f/f* mice show increased distance in N36 *vs.* N1, but no changes *vs.* N14, likely due to lower peak values (as compared with WTs) rather than improved memory of acquired skills.

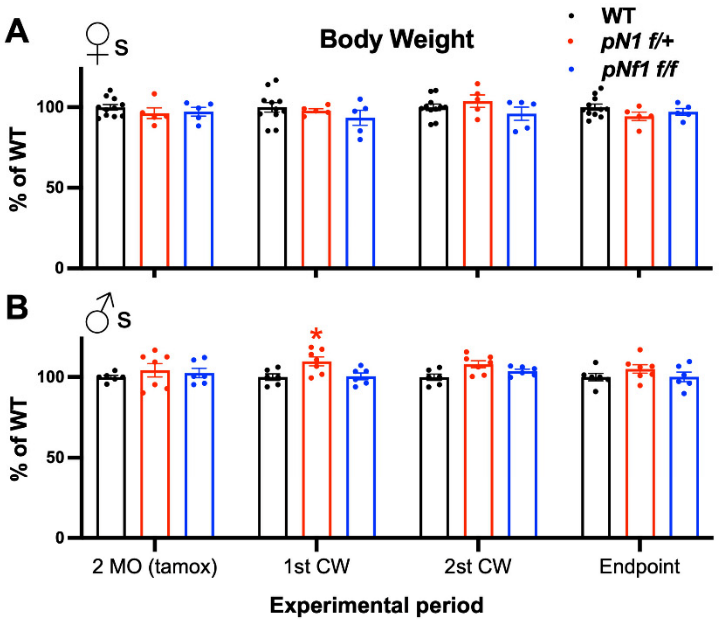

Supplementary Figure S2. **No overall impact of *pNf1* mutation on body weight**. The body weight of female (A) and male (B) WT (black), *pNf1f/+* (red) and *pNf1f/f* (blue) mice was recorded at 4 key time points of the experiment: at 2 months old (2MO) just before tamoxifen administration; at the time of the first introduction to CWs (2-6 months after tamoxifen treatment); at the time of the re-introduction to CWs (after a 3 -week break without wheels); and at the end of the CW test. Data are shown as percent of WTs for each experimental period. Two-way ANOVA, Bonferroni's post-hoc tests with genotype as source of variation (*p<0.05).

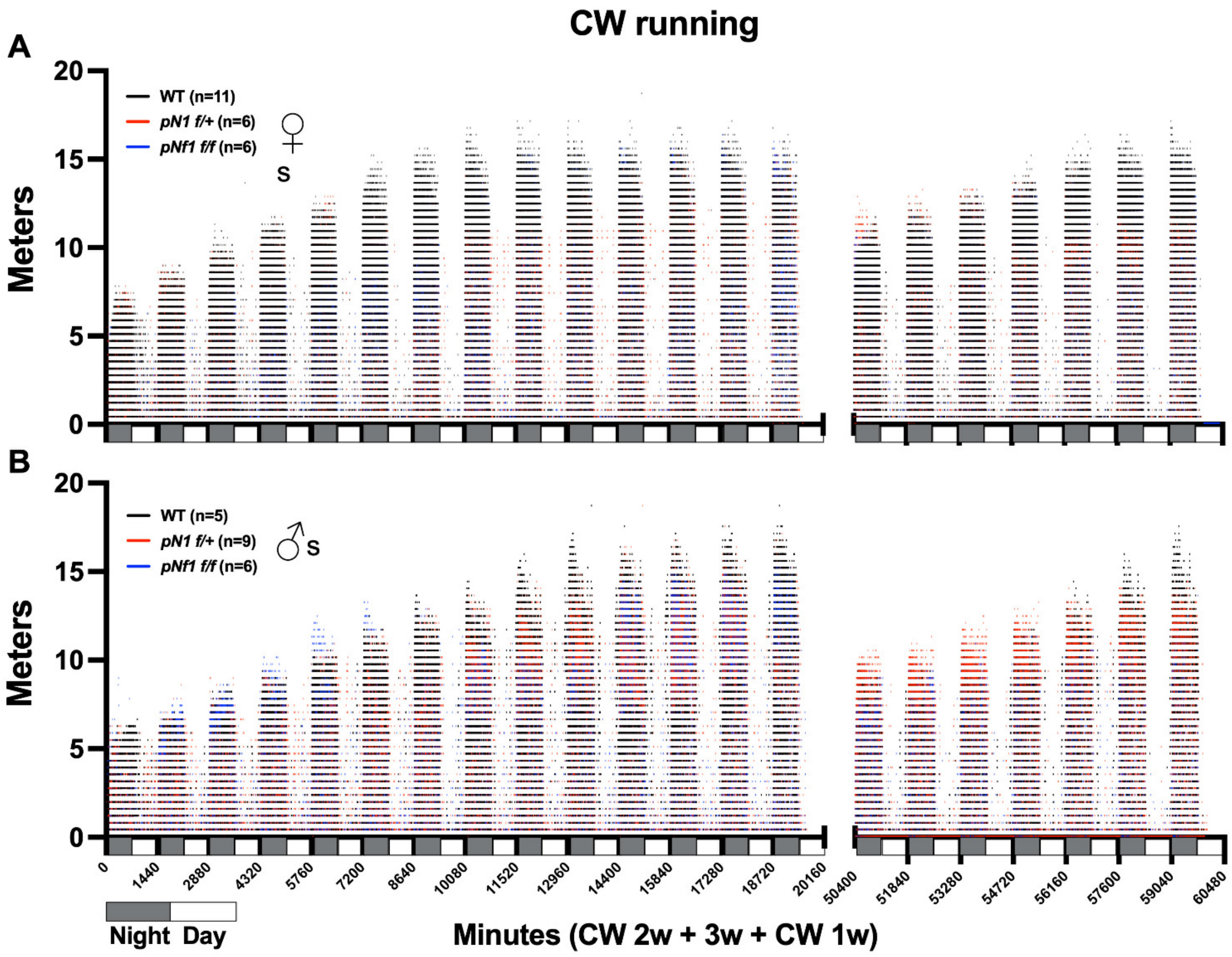

Supplementary Figure S3. **No overall impact of *pNf1* mutation on nocturnal running activity in the CW test**. Female (top) and male (bottom) WT (black), *pNf1f/+* (red), and *pNf1f/f* (blue) mice were subjected to the CW test: mice were introduced to cages with CWs for 2 weeks, were housed without wheels for 3 weeks, and re-introduced to CWs for an additional week. Distance run every minute was plotted for the entire experiment. Note the marked nocturnal activity (dark shaded vs. open box for days) in all genotypes.

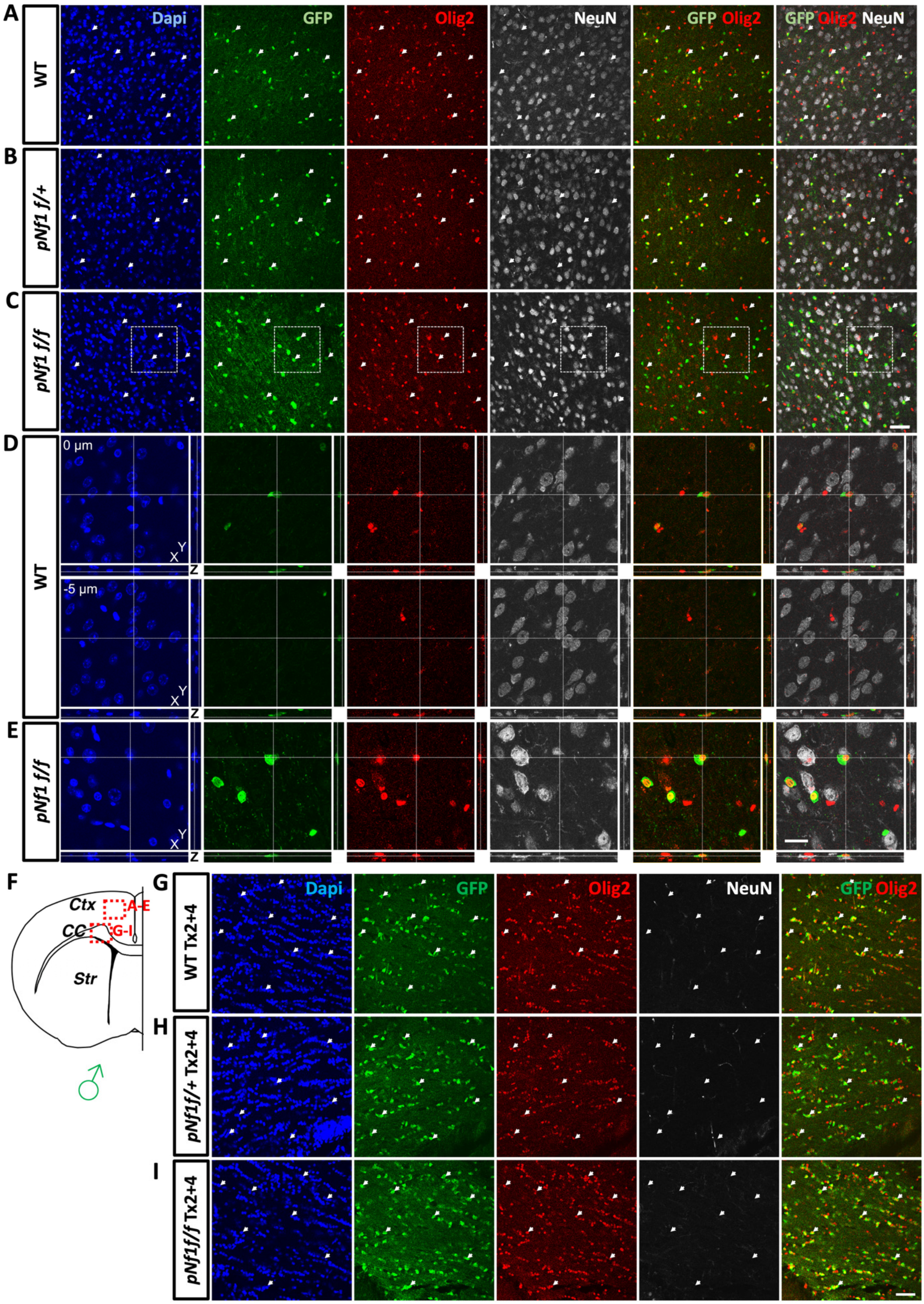

Supplementary Figure S4. **Recombination driven by *PlpCre*^{ER} is highly specific for OL lineage cells in the motor cortex and CC**. Brain sections containing the Cortex (A-E) and corpus callosum (CC; G-I, diagram in F) of WT (A, D), *pNf1f/+* (B), and *pNf1f/f* (C, E) mice subjected to CWs, immunostained to detect

the reporter EGFP (green), the OL-lineage marker Olig2 (red), and the neuronal marker NeuN (white). Cell nuclei are labeled with DAPI (blue). Arrows indicate EGFP+ recombinant cells co-immunodetecting Olig2+ but not NeuN+ signals. D) High magnification orthogonal projection showing two Z axis levels of the same x/y areas in a WT mouse; 0μm level shows an EGFP+,Olig2+,NeuN- recombinant cell while level -5μm shows an EGFP-,Olig2-,NeuN+ neuron. E) High magnification orthogonal projection showing the area indicated in C (doted square), depicting EGFP+Olig2+ cells in close proximity to, but independently from, NeuN+ cells. A-C, G-I scale bar = 50μm, D-E scale bar = 20μm.

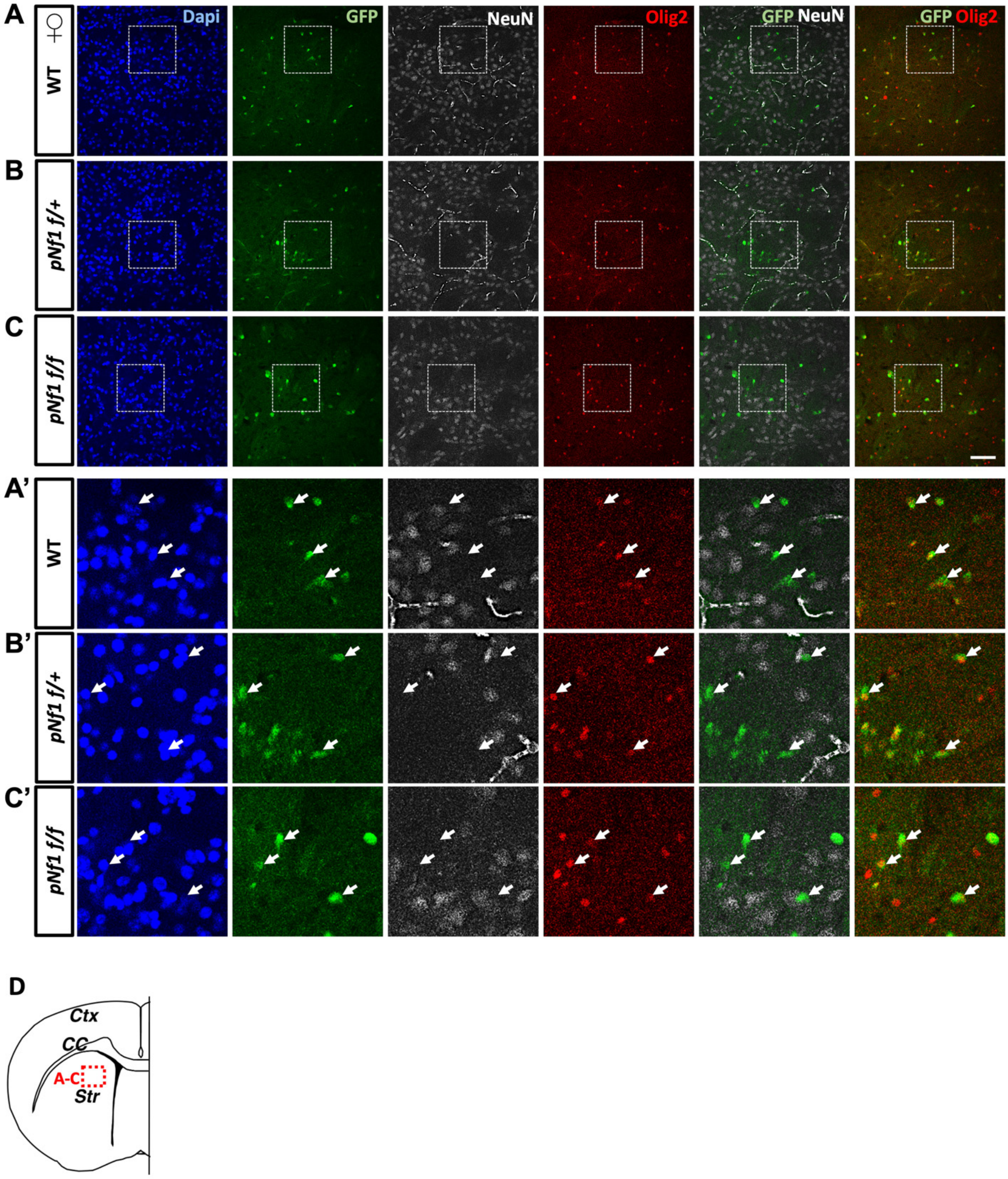

Supplementary Figure S5. **Recombination driven by *PlpCre^{ER}* is highly specific for OL lineage cells in the Striatum**. Brain sections containing the Striatum of WT (A), *pNf1f/+* (B), and *pNf1f/f* (C) mice (diagram in D) subjected to CWs, immunostained to detect the reporter EGFP (green), the OL-lineage marker Olig2 (red), and the neuronal marker NeuN (white). Cell nuclei are labeled with DAPI (blue). A'-C') Higher

magnification from A-C indicating EGFP+ recombinant cells co-immunodetecting Olig2+, in close interaction but not co-immunodetecting NeuN+ signals. Scale bar = 50µm.

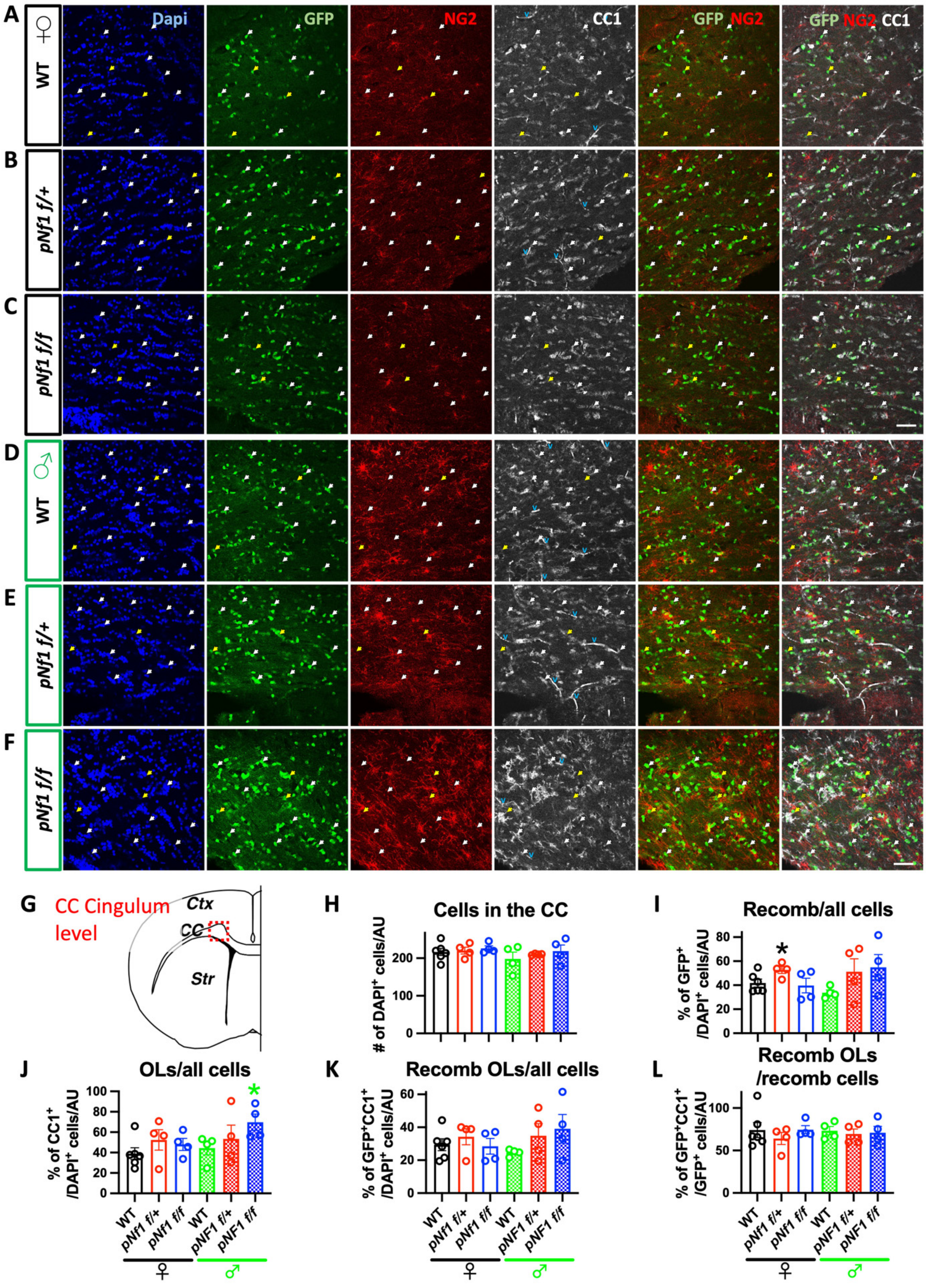

Supplementary Figure S6. **Density and fate of recombined cells is comparable in the CC of WT and *pNf1* mice**. Female (A-C) and male (D-F) brain sections containing the corpus callosum (CC, cingulum level, diagram in G) of WT (A, D), *pNf1f/+* (B, E), and *pNf1f/f* (C, F) mice subjected to CWs, immunostained to

detect the reporter EGFP (green), the Oligodendrocyte Progenitor (OPC) marker NG2 (red), and the OL marker CC1 (white). Cell nuclei are labeled with DAPI (blue) and unspecific signals in blood vessels are pointed with “v” (cyan). Arrows indicate EGFP+ recombinant cells co-immunodetecting with CC1 but not with NG2. Scale bar = 50µm. Cell quantification of all DAPI+ cells (H), % of EGFP+ recombinant cells among DAPI+ cells (I), CC1+ OLs among DAPI+ cells (J), EGFP+CC1+ recombinant OLs among DAPI+ cells (K), and EGFP+CC1+ recombinant OLs among recombinant cells (L). Student's *t* test: *p < 0.05 (n = 4 mice/genotype/gender). Data are the mean + SEM.

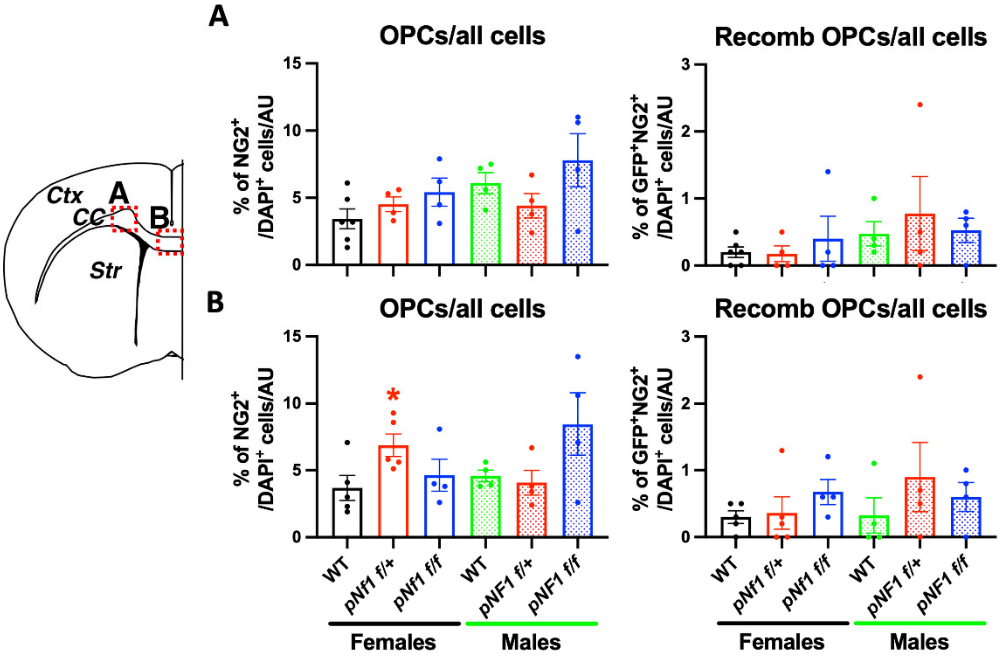

Supplementary Figure S7. **Non-cell-autonomous response of OPCs in the CC of *pNf1* mice**. Quantification of immunostained NG2+ (left) and NG2+EGFP+ (right) cells (representative images in Fig. 5) as percentage of all cells (DAPI+) in equivalent areas of the midline (A) and cingulum (B) levels of the CC (left diagram). Color code: males (empty bars), females (pattern-filled bars), WT (females black; males green), *pNf1f/+* (red), and *pNf1f/f* (blue). Female *pNf1f/+* mice show increased number of NG2+ OPCs (B, left bottom); however, the density of recombinant OPCs remains is <1% and without changes in *Nf1* mutants

(right), indicating a non-cell-autonomous nature of the phenotype. Student's *t* test: *p < 0.05 (n = 4 mice/genotype/gender), data are the mean + SEM.

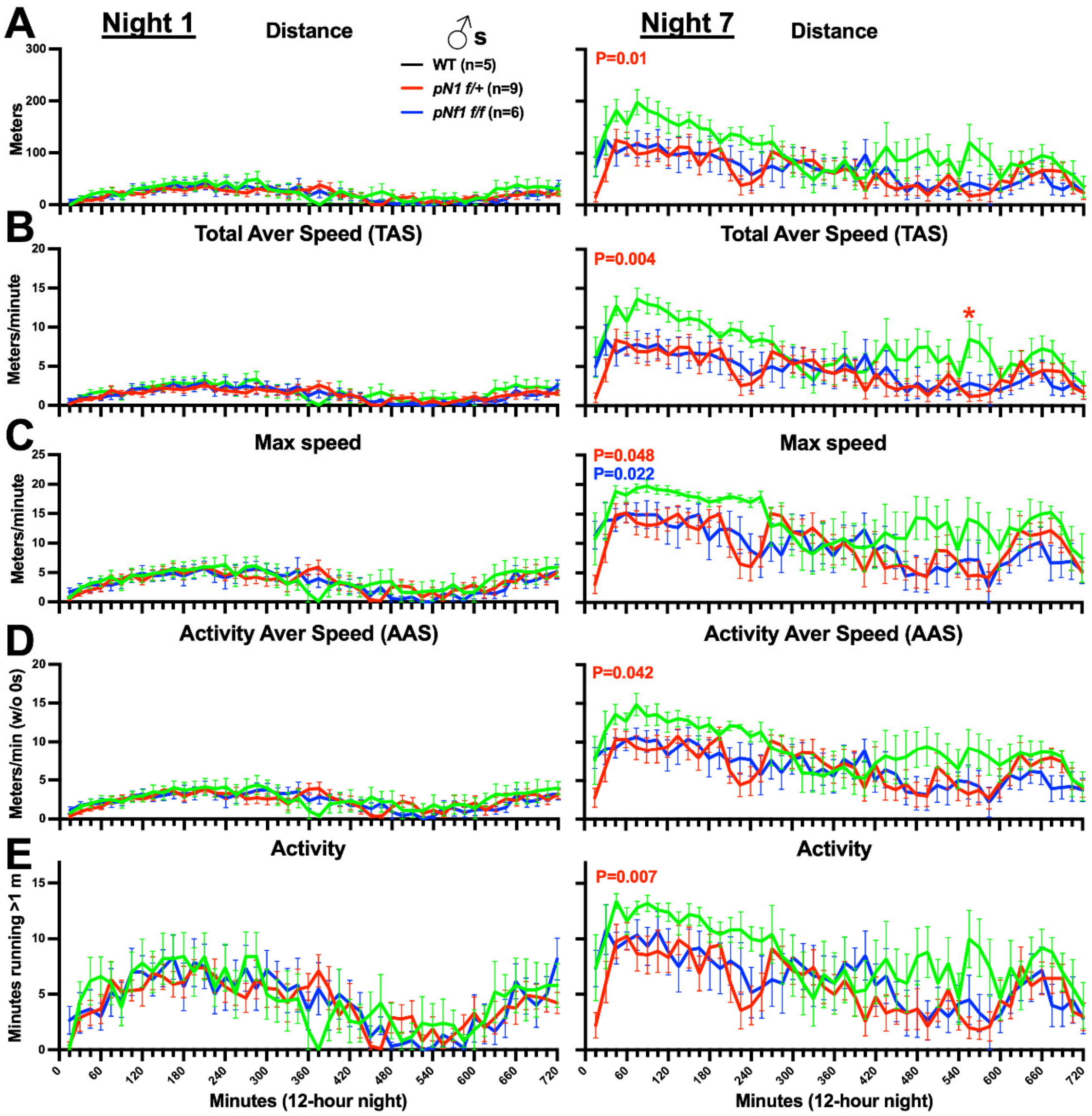

Supplementary Figure S8. **Progressive CW running issues from N1 to N7 in *pNf1* mutant males**. A-E) Raw data from night 1 (N1, left) and night 7 (N7, right) of the first introduction to CWs were divided in 15-minute intervals, and CW parameters for Wild Type (green), *pNf1f/+* (red), and *pNf1f/f* (blue) males were plotted: A) total distance run, B) Total Average Speed (TAS), C) Max speed, D) Activity Average Speed (AAS), and E) activity. Significant P values from two-way ANOVA tests with genotype as source of variation (WT *vs. pNf1 f/+*; red, WT *vs. pNf1 f/f*; blue) are shown for each plot. Significant differences from Individual night comparisons for WT vs. *pNf1f/+* and WT *vs. pNf1f/f* (Bonferroni's post-hoc tests, *p<0.05) are shown.

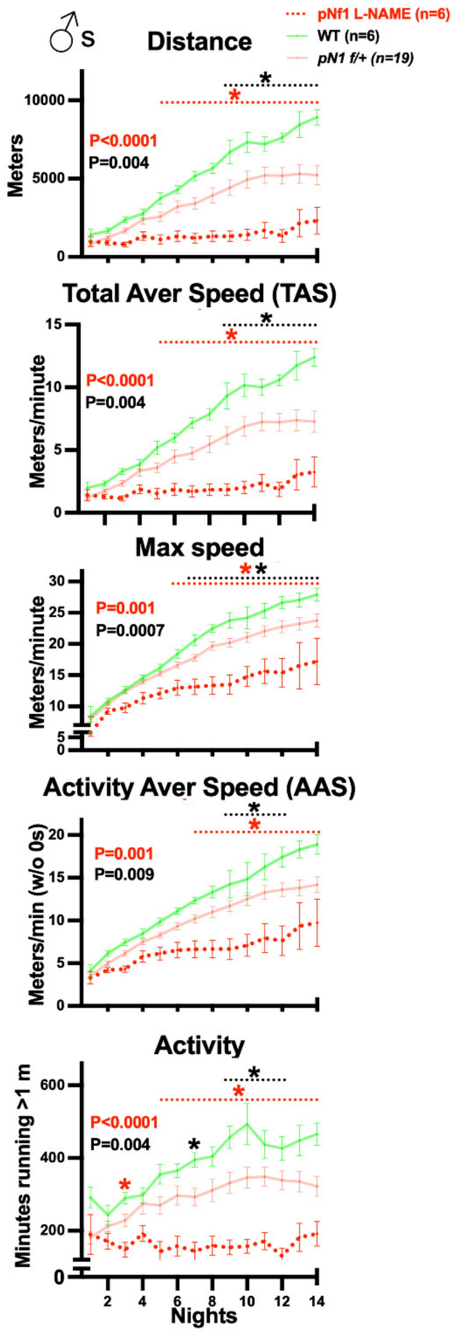

Supplementary Figure S9. **L-NAME treatment negatively impacts learning curves in *pNf1* males.** X axis labels are shared in A-E. Plots for nightly values of CW parameters for *pNf1 f/+* male mice treated with L-NAME (0.3 mg/L in drinking water): A) total distance (meters), B) Total Average Speed (TAS), D) max speed, E) Activity Average Speed (AAS), and F) minutes with activity. Plots for untreated WT and *pNf1* females are shown in faded colors as control values (compare with Fig. 3). Statistically significant P values (two-way ANOVA test, treatment as source of variation) are shown (*P red: L-NAME treated *pNf1 f/+ vs.* untreated WT,

*P black: L-NAME treated *pNf1 f/+* *vs.* untreated *pNf1 f/+* mice. Comparison of individual nights in are also shown (same color code, Bonferroni's post-hoc tests, *P<0.05).
